# Human Gut Symbiont *Roseburia hominis* Promotes and Regulates Innate Immunity

**DOI:** 10.3389/fimmu.2017.01166

**Published:** 2017-09-26

**Authors:** Angela M. Patterson, Imke E. Mulder, Anthony J. Travis, Annaig Lan, Nadine Cerf-Bensussan, Valerie Gaboriau-Routhiau, Karen Garden, Elizabeth Logan, Margaret I. Delday, Alistair G. P. Coutts, Edouard Monnais, Vanessa C. Ferraria, Ryo Inoue, George Grant, Rustam I. Aminov

**Affiliations:** ^1^Rowett Institute of Nutrition and Health, University of Aberdeen, Aberdeen, United Kingdom; ^2^INSERM, UMR1163, Lab Intestinal Immunity, Paris, France; ^3^Université Paris Descartes-Sorbonne Paris Cité and Institut Imagine, Paris, France; ^4^Micalis Institute, INRA, AgroParisTech, Université Paris-Saclay, Jouy-en-Josas, France; ^5^Kyoto Prefectural University, Kyoto, Japan; ^6^School of Medicine, Medical Sciences and Nutrition, University of Aberdeen, Aberdeen, United Kingdom; ^7^Institute of Fundamental Medicine and Biology, Kazan Federal University, Kazan, Russia

**Keywords:** *Roseburia*, T lymphocytes, immune tolerance, inflammatory bowel disease, flagellin, TLR5

## Abstract

**Objective:**

*Roseburia hominis* is a flagellated gut anaerobic bacterium belonging to the *Lachnospiraceae* family within the Firmicutes phylum. A significant decrease of *R. hominis* colonization in the gut of ulcerative colitis patients has recently been demonstrated. In this work, we have investigated the mechanisms of *R. hominis*–host cross talk using both murine and *in vitro* models.

**Design:**

The complete genome sequence of *R. hominis* A2-183 was determined. C3H/HeN germ-free mice were mono-colonized with *R. hominis*, and the host–microbe interaction was studied using histology, transcriptome analyses and FACS. Further investigations were performed *in vitro* and using the TLR5KO and DSS-colitis murine models.

**Results:**

In the bacterium, *R. hominis*, host gut colonization upregulated genes involved in conjugation/mobilization, metabolism, motility, and chemotaxis. In the host cells, bacterial colonization upregulated genes related to antimicrobial peptides, gut barrier function, toll-like receptors (TLR) signaling, and T cell biology. CD4^+^CD25^+^FoxP3^+^ T cell numbers increased in the *lamina propria* of both mono-associated and conventional mice treated with *R. hominis*. Treatment with the *R. hominis* bacterium provided protection against DSS-induced colitis. The role of flagellin in host–bacterium interaction was also investigated.

**Conclusion:**

Mono-association of mice with *R. hominis* bacteria results in specific bidirectional gene expression patterns. A set of genes thought to be important for host colonization are induced in *R. hominis*, while the host cells respond by strengthening gut barrier function and enhancing Treg population expansion, possibly *via* TLR5-flagellin signaling. Our data reveal the immunomodulatory properties of *R. hominis* that could be useful for the control and treatment of gut inflammation.

## Introduction

The human gut microbiota consists of more than 500–1,000 different phylotypes the majority of which belong to the Bacteroidetes and Firmicutes bacterial phyla (1). Successful symbiotic relationships arising from bacterial colonization of the human gut yield a wide variety of metabolic, structural, protective, and other beneficial functions. The immunological importance of the gut microbiota is also well recognized; it is particularly apparent in germ-free (GF) animals that have an impaired immune system, which, however, can be functionally reconstituted by the introduction of gut commensal bacteria (2–4).

In sharp contrast to the production of secretory intestinal IgA, which is mainly driven by microbial colonization *per se* (5, 6), the development and differentiation of T cells require colonization by specific commensal bacteria. Some species of clostridia, such as segmented filamentous bacteria (SFB), appear to be potent inducers of differentiation and maturation of intestinal Th1, Th17, and Treg cell lineages (7, 8). Recent studies have demonstrated that the clostridia clusters IV and XIVa and the Altered Schaedler Flora can induce *de novo* generation of Treg cells, while mono-colonization with *Bacteroides fragilis* can correct the Th1/Th2 imbalance in germ-free mice by promoting the expansion of Treg cells (4, 9, 10). The effects of commensal bacteria on T cell differentiation pathways are variable and may be influenced by a specific array of toll-like receptors (TLR) ligands associated with particular bacteria (11). For instance, the Treg-enhancing effects of *B. fragilis* are implemented *via* TLR2 signaling by polysaccharide A (12).

Dramatic changes in microbiota composition affecting the balance of symbionts and pathobionts have been documented in gastrointestinal disorders such as inflammatory bowel disease. Crohn’s disease (CD), for example, is characterized by a greater relative abundance of the Proteobacteria and a reduction of other bacteria such as the Bacteroidales and Clostridiales (13–17). A specific decrease of *Roseburia* spp. in patients with CD has also been noted (16, 18). Interestingly, this microbial dysbiosis is also associated with imbalances in T effector cell populations. The gut microbiota alterations in ulcerative colitis (UC) have not been characterized to the same extent. Only recently, a study of UC patients has demonstrated that *Roseburia hominis*, together with *Faecalibacterium prausnitzii*, is significantly decreased in this disease (16, 19). Both species display an inverse correlation with disease activity. Previous studies have shown anti-inflammatory effects of *F. prausnitzii* colonization on the host (20). Much less is known about *R. hominis*, which is a member of the clostridia cluster XIVa.

The complete DNA sequence and annotation of the *R. hominis* genome has been previously described (21). In this paper, bacterial and host transcriptome responses to *R. hominis* colonization were investigated. Bacterial responses included expression of genes involved in colonization of and adaptation to the environment of the murine gut, while host responses to colonization included genes of immunity and gut function.

## Materials and Methods

### Bacterial Cultures

*Roseburia hominis* A2-183^T^ (=DSM 16839^T^ = NCIMB 14029^T^) and other *Roseburia* species were maintained and grown on synthetic YCFA media as described before (22). *Escherichia coli* and *Salmonella enterica* strains were cultivated in LB medium. All *Roseburia* culture manipulations were performed in a MACS-MG-1000 anaerobic workstation (Don Whitley Scientific) under an atmosphere of 80% N_2_, 10% CO_2_ and 10% H_2_ at 37°C.

### Animals, Experimental Design, and Sampling

Germ-free animal experiments were performed in the gnotobiotic rodent breeding facility of INRA (ANAXEM platform, Institut Micalis, INRA, Jouy-en-Josas, France). The GF C3H/HeN male mice were allocated into the control (*N* = 8) and treatment (*N* = 10) groups and caged individually. *R. hominis* A2-183^T^ (=DSM 16839^T^ = NCIMB 14029^T^) was grown anaerobically at 37°C in YCFA media. At days 0, 1, and 2, animals in the treatment group were given 10^9^ colony-forming units (CFU) of *R*. *hominis* culture by gavage, while control animals were given 100 µL of YCFA medium. The ileal, ascending colonic, and cecal samples were collected at days 14 and 28. GF TLR5KO (C57BL/6 genetic background, *N* = 3) and C57BL/6 (*N* = 3) animals (Jouy-en-Josas), and conventional TLR5KO (C57BL/6 genetic background, *N* = 6) and Boy/J (C57BL/6 congenic, *N* = 6) animals (Medical Research Facility, University of Aberdeen) were gavaged with the live cultures of *R. hominis* to evaluate its functional importance. Boy/J (C57BL/6 B6 Cd45.1) mice were the background controls for conventional TLR5K0. They carry a CD45.1 pan leukocyte marker but are otherwise equivalent to C57BL/6 wild-type mice (23, 24). Twelve female C57BL/6 (6 weeks old) mice were used to evaluate the effect of *R. hominis* during DSS-induced mild colitis. Three mice were dosed daily by direct administration of a culture of *R. hominis* for a total of 8 days. Six mice were dosed with culture medium. Exposure to DSS was for 4 days (from day 3 to day 6). Three *R. hominis*-dosed and three control mice were offered sterile water containing DSS (20 g/L) over this period. On day 7, these mice were switched back to sterile water with no DSS. The mice were euthanased and dissected aseptically on day 9. Twenty-two female C57BL/6 mice (6 weeks old) were used to evaluate the effect of *R. hominis* during DSS-induced pathological colitis. After the acclimatization period of 7–10 days, the mice were dosed daily with 50 µL (10^9^ CFU) of *R. hominis* in growth culture medium for 14 days. Control animals were given growth culture medium. From day 9, mice were given DSS (MW 50 kDa, 30 g/L) in their drinking water for 6 days. The animals were euthanized on day 15 and tissue sampling was performed as described earlier. The management and experimental procedures with animals were approved by the respective Local Ethical Review Committees.

### Transcriptome Analyses

Bacterial RNA from the mouse cecum contents was isolated and labeled with dCTP-Cy3 or dCTP-Cy5 during cDNA synthesis. PCR products amplified from an *R. hominis* small fragment library (6,000 clones) were used to create duplicate microarrays on glass slides using a MicroGrid II TAS array-spotting robot (BioRobotics). Microarray hybridization was performed in a GeneTAC hybridization station (Genomic Solutions). Dye-swap and separate RNA purification protocols were used to avoid potential biases.

Murine RNA was extracted from the ileum and ascending colon tissues and hybridized to the GeneChip “NuGO Mouse Array” and GeneChip “Mouse Genome Array” (Affymetrix). Data analysis was performed using R^1^ and Bioconductor.^2^ The microarray data were submitted to NCBI GEO (Gene Expression Omnibus) with accession number GSE25544.

The *R. hominis*-specific primers 5′-CCCACTGACAGAGTATGTAATGTAC-3′ and 5′-GCACCACCTGTCACCAC-3′ were used for qPCR analyses of fecal samples to validate the efficiency of colonization. The analyses were performed using a 7500 Fast Real-Time PCR System (Applied Biosystems) with a Power SYBR Green PCR Master Mix (Applied Biosystems). RT-qPCR analyses were performed on the ileal and colonic RNA samples for host gene expression studies using a QuantiFast SYBR Green PCR Kit (Qiagen) and QuantiTect Primer Assays (Qiagen).

### Immunofluorescence and Histology

FISH analyses were performed with neutral buffered, formalin-fixed gut tissue sections (2 µm thick) with 16S rRNA probes, specific for the domain of Bacteria (Cy3-labeled Eub338) and specific for the *R. hominis* strain A2-183 (FITC-labeled GTACATTACATACTCTGTCAGTG). Bacteria were visualized at x630 magnification. Immunolocalization of *R. hominis* flagellin was examined in methanol-fixed colon content smears using rabbit antisera anti-FlaA1 or anti-FlaA2 (Covalab) and Alexa donkey anti-rabbit 488 (Molecular Probes). T cell markers were examined on sequential intestinal tissue sections (8 µm) using Ly6G-FITC, CD3-FITC, isospecific IgG (BD Biosciences), and double-labeled FoxP3 Alexa Fluor 594 (Abcam) and CD3-FITC. Intestinal tissue sections (4 µm) were also stained with hematoxylin/eosin. A complete transverse cross-section of the colon from each animal was visualized at ×200 magnification. Each field of view was scored from 0 to 4 according to the method based on Ref. (25).

### Cell Experiments

Caco-2 and HT29 cells were grown at 37°C in a 75% humidified atmosphere with 5% CO_2_ in 24-well plates. Before any treatment, cells were washed twice with Hanks’ Balanced Salt Solution and kept in DMEM supplemented with l-glutamine, selenium, and transferrin for 24 h. Epithelial cells were harvested after incubation for 2 or 4 h and then stored in RNAlater for further analyses.

Bone marrow cells were harvested from the femur and tibia of C3H/HeN and C57BL/6 mice. Bone marrow-derived dendritic cells (BMDCs) were generated by Flt3L or GM-CSF culture, stimulated with 100 ng/mL flagellin, and analyzed by flow cytometry. Supernatants were collected and tested for IL-10 and IL-12 by cytometry bead arrays (BD Bioscience).

Cells from the intestine and mesenteric lymph nodes (MLNs) were isolated as described previously (26), with minor modifications. In brief, cell suspensions were incubated with 100 U/mL of collagenase VIII (Sigma-Aldrich) in RPMI supplemented with 20% of FBS at 37^o^C for 20 min (MLN) or 60 min (intestine). Single cell suspensions were analyzed by flow cytometry. For mixed leukocyte reactions, naïve CD4^+^ T cells were purified from the major lymph nodes and spleens of OTII transgenic mice using MACs Miltenyi CD4^+^ T cell Isolation Kit as per manufactures instruction. Purified CD4^+^ T cells were co-incubated with flagellin activated Flt3L-derived BMDCs in the absence or presence of OVA323–339 peptide (1 µg/ml). After 5 days, T cell differentiation was determined by flow cytometry.

### Flow Cytometry

Intestinal lamina propria cells and MLN cells were labeled with CD4-FITC, CD25-APC (eBioscience), CD8-APC-Cy7, CD3-PerCP (Biolegend), and B220-BV570 (BD Biosciences). Labeling of intracellular FoxP3 was performed after extracellular staining and fixation/permeabilization of cells. GM-CSF-derived dendritic cells were labeled with CD11b-PerCP Cy5.5 (BD Biosciences), CD11c-PE-Cy7, I-A/I-E-APC-Cy7, CD80-PE, CD86-APC, CD8-FITC, and B220-BV570 (Biolegend). Flt3L-derived dendritic cells were labeled with CD11c-PE-Cy7, CD11b- or Siglec-H-PerCP Cy5.5 (Biolegend), I-A/I-E-APC-Cy7, CD317-PE, CD40-Alexa Fluor 647, CD103-FITC, and B220-BV570. Coculture T cells were labeled with CD4-PerCP Cy5.5 (BD Biosciences), CD127-PE-Cy7, CD73-APC, CD195 (CCR5)-FITC, CD62L-BV570, and CD25-APC-Cy7 (Biolegend).

### Statistical Analyses

Spot intensities on the microarrays were log-transformed and Loess normalization was applied to the microarray results. RT-qPCR data were transformed on a base-2 logarithmic scale and analyzed by one-way analysis of variance with a significance threshold of *P* < 0.05. Statistical significance between the treatment groups was evaluated using Student’s *t*-test.

More detailed protocols are available in SI Materials and Methods in Supplementary Material.

## Results

### *R. hominis* Responds to the Gut Environment by Upregulating Chemotaxis, Motility, and Mobilization Genes

**Figure 1:**
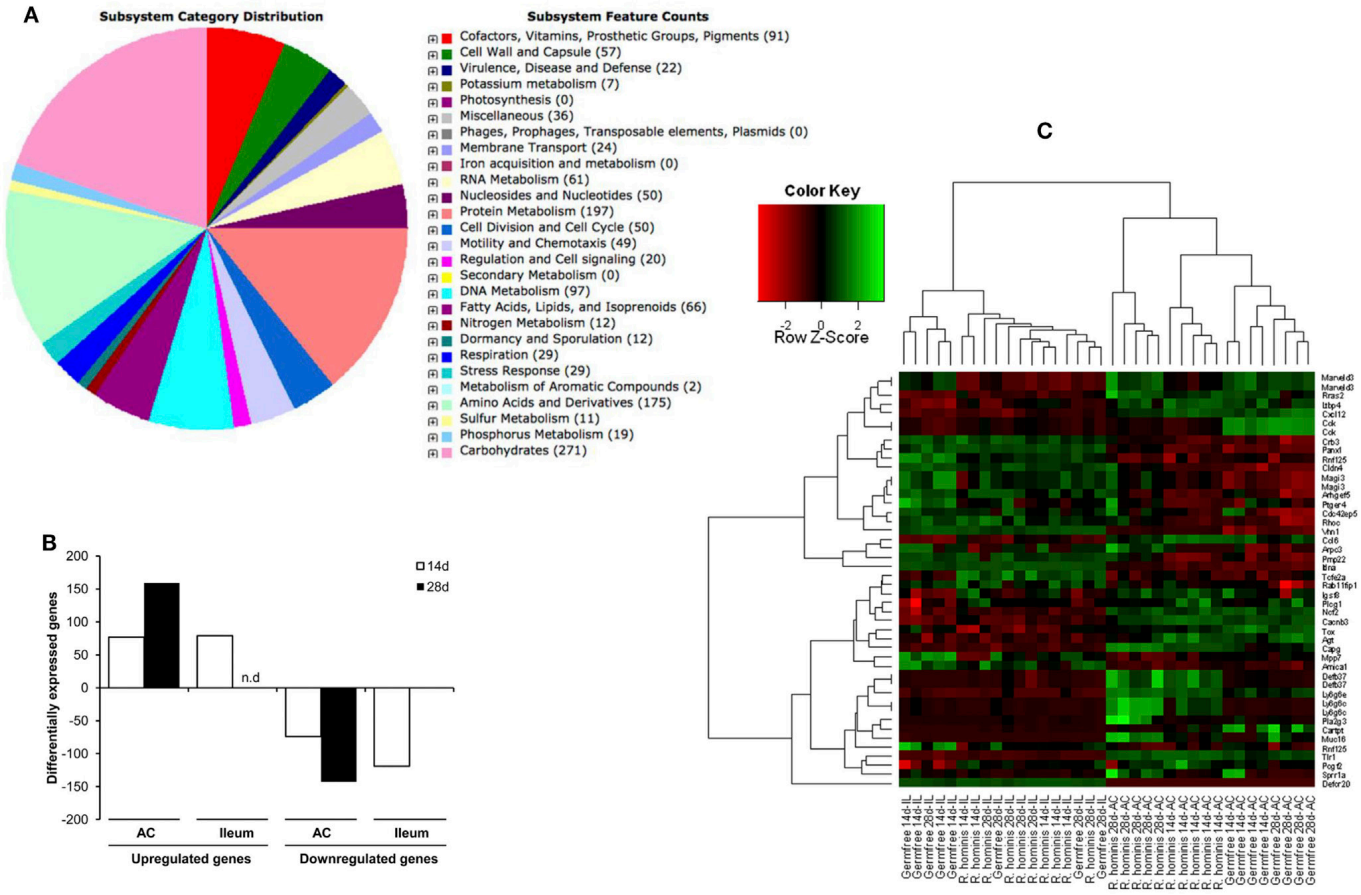
Sequence features of *Roseburia hominis* genome and differentially expressed transcripts in the murine gut after mono-association with *R. hominis*. **(A)** Subsystem category distribution in the genome of *R. hominis* according to RAST. The largest subsystem feature counts belong to carbohydrates, amino acids and derivatives, and protein metabolism. **(B)** The number of differentially expressed genes in *R. hominis*-colonized mice (*N* = 5) compared to germ-free (GF) control (*N* = 4) identified with the Affymetrix microarray. Bar graphs represent the number of genes up- or downregulated in mice after 14 and 28 days of colonization.**(C)** Heatmap generated from differentially expressed genes with functional significance between GF and *R. hominis*-colonized mice at 14 and 28 days.

Genome analysis (Figure 1A) identified about 5% of all genes were related to chemotaxis and motility function including four different flagellin genes of which one located within the flagellar operon is associated with flagellar motility.

A biomass of *R. hominis* was given by gavage to GF C3H/HeN mice on three consecutive days. *R. hominis*-colonized both the ileum and colon but was found in much higher numbers in the colon, up to 1 × 10^10^ bacteria/g feces (Figure S1 in Supplementary Material) and was found to be closely associated with the colonic mucosa.

Differential gene expression in the bacterium in response to association with the host was investigated using the *R. hominis* microarray. Bacterial RNA was isolated from three different experimental conditions to distinguish between the effects of the gut environment and animal dietary components, (i) *in vivo*, from the cecum of mono-associated mice; (ii) *in vitro*, from bacteria grown in the presence of dietary components; and (iii) *in vitro*, from bacteria grown in culture.

Colonization of GF mice with *R. hominis* correlated with the increased host gene expression, which was highest in the colon, particularly at day 28 post-colonization (Figure 1B). The number of differentially expressed genes in the ileum was very low at day 28, consistent with the reduced bacterial numbers. Differential transcriptome responses in the two tissue sites and time points were illustrated by a clear separation of significant transcripts in the heatmap analysis (Figure 1C).

**Figure 2:**
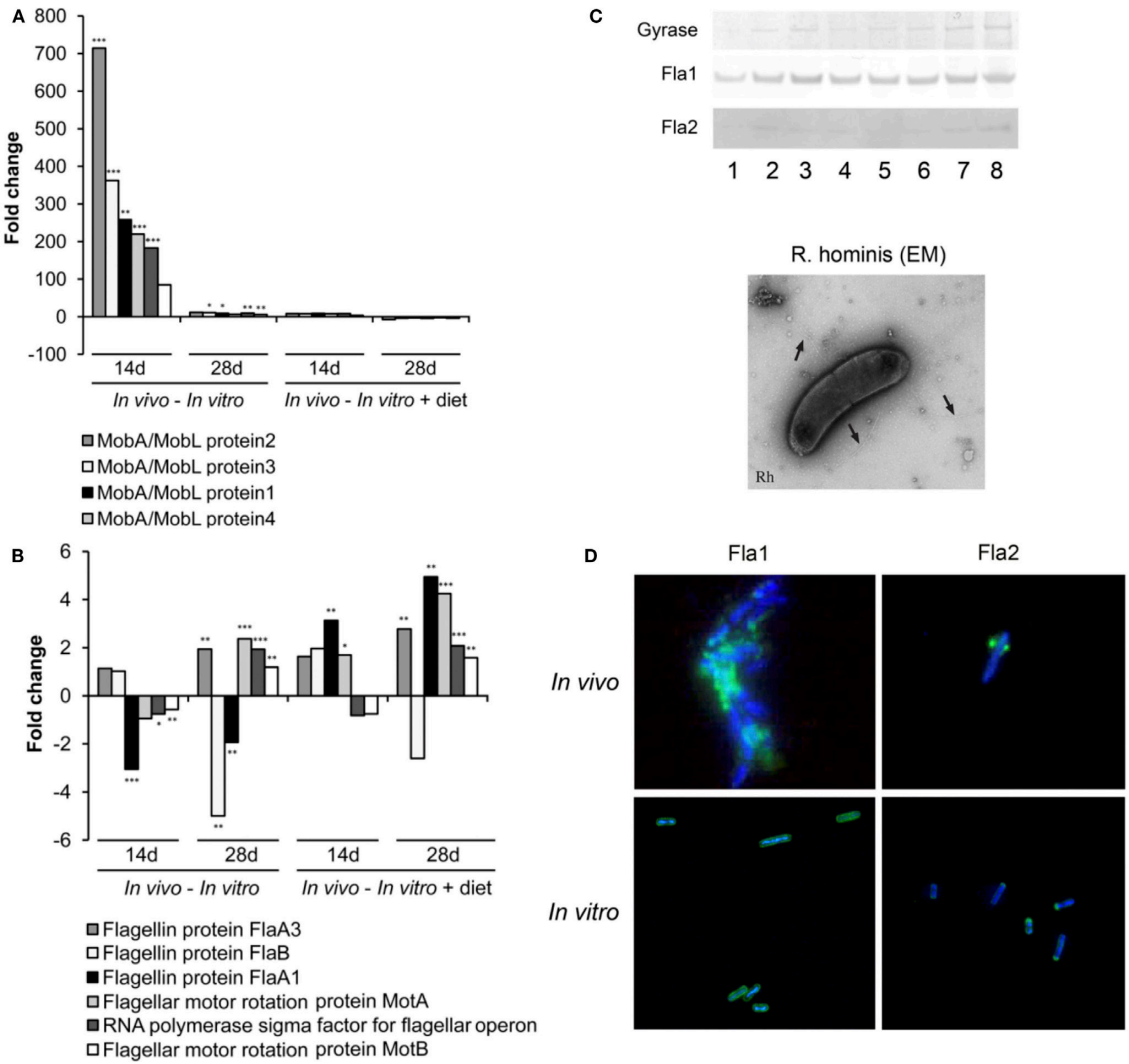
Responses of *Roseburia hominis* to the gut environment. **(A)** RT-qPCR quantification of *R. hominis* transcripts involved in conjugation/mobilization transfer. *R. hominis* RNA samples: *in vivo*—isolated from mono-associated animals; *in vitro*—isolated from YCFA medium-grown cultures; *in vitro* + diet—isolated from cultures grown on YCFA medium with addition of murine chow. **(B)** RT-qPCR quantification of *R. hominis* transcripts involved in motility and chemotaxis. *R. hominis* RNA samples: *in vivo*—isolated from mono-associated animals; *in vitro*—isolated from YCFA medium-grown cultures; *in vitro* + diet—isolated from cultures grown on YCFA medium with addition of murine chow. **(C)** Western blot of *R. hominis* proteins grown *in vitro* in the presence of UV-irradiated standard murine chow. The membrane was immunostained with affinity-purified 1 antibody, Fla2 specific antiserum, and anti-DNA gyrase A antibody (lane 1: no diet, lane 2: 0.01 g diet/10 mL of *R. hominis* culture, lane 3: 0.02 g diet/10 mL, lane 4: 0.05 g diet/10 mL, lane 5: 0.1 g diet/10 mL, lane 6: 0.2 g diet/10 mL, lane 7: 0.5 g diet/10 mL, and lane 8: 1 g diet/10 mL). Electron microscopy of *R. hominis* showing flagella (black arrows). **(D)** Immunocytochemistry with FlaA1 and FlaA2 specific antisera performed on *R. hominis* from the luminal contents of mono-colonized mice and on *R. hominis* grown *in vitro*. Original magnification is ×1,000. RT-qPCR results are presented as fold change. Statistical significance: *P < 0.05, **P < 0.01, and ***P < 0.001.

Differentially expressed genes were identified *(in vivo* vs*. in vitro*) with RT-qPCR validation performed on 42 differentially expressed genes (Tables S1 and S2 in Supplementary Material). The *mobA*- and *mobL*-like genes that are involved in conjugation/mobilization transfer showed strong upregulation *in vivo* (Figure 2A). Other subsystems induced by the gut environment included membrane transport, in particular magnesium transport, and motility and chemotaxis including multiple methyl-accepting chemotaxis proteins and genes of the flagellar operon (Figure 2B). *R. hominis* possesses multiple flagellin genes: *flaA1, flaA2, flaA3*, and *flaB*. The expression of *flaA1*and *flaA2* in the murine gut environment was verified by Western–blot and immunocytochemistry (Figure 2C).

### *R. hominis* Affects Host Innate Signaling Pathways

Genes involved in innate immunity and gut barrier function of the host were significantly induced by *R. hominis* colonization [Figure 1C; NCBI GEO (Gene Expression Omnibus), accession number GSE25544]. The GO process “innate immune response” (GO:0045087) was upregulated and included the TLR-related genes *Tlr5, Tlr1*, and *Vnn1*. The upregulation of *Tlr5* was of particular interest, given the presence of flagellar genes in the genome of *R. hominis* and the expression of the corresponding proteins in the gut of mono-colonized mice. The flagellins may have a role in this innate signaling pathway to mediate innate and adaptive immune responses. Other innate immunity genes affected by *R. hominis* colonization included the antimicrobial peptides *Defb37, Pla2g3, Muc16*, and *Itln* and the gut barrier function genes *Sprr1a, Cldn4, Pmp22, Crb3*, and *Magi3*. Innate immunity genes that were upregulated in the ileum in response to *R. hominis* included *Defcr20, Pcgf2, Ltbp4, Igsf8*, and *Tcfe2a*. We also found a negative regulation of the NF-κB pathway (GO:0043124) by *R. hominis*, which may contribute to the immune homeostasis by downregulating this inflammatory cascade.

### *R. hominis* Directs T Cell Pathways

**Figure 3:**
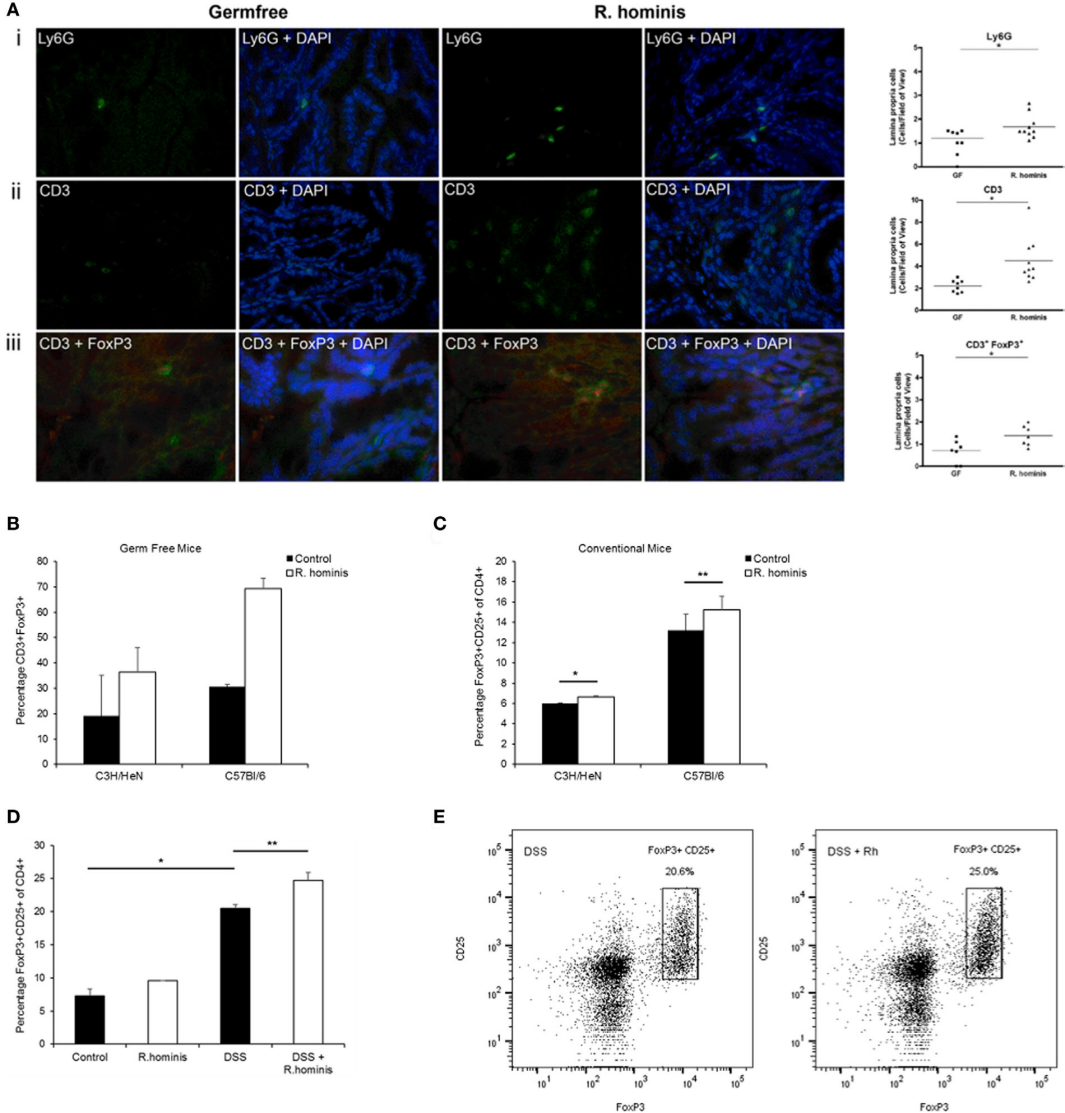
Induction of FoxP3^+^ Treg cells by *Roseburia hominis*. **(A)** Immunofluorescence analysis of lamina propria cells labeled with (i) anti-Ly6G, (ii) anti-CD3, and (iii) anti-CD3/anti-FoxP3 in the ascending colon of germ-free (GF) and *R. hominis*-treated C3H/HeN mice. Data are shown as the number of positive cells per field of view in GF (*N* = 7–8) and *R. hominis*-treated mice (*N* = 8–10). Original magnification ×630. Statistical significance: *P < 0.05. **(B)** Immunofluorescence analysis of lamina propria cells in the ascending colon labeled with anti-CD3 and anti-FoxP3 in germ-free and *R. hominis* mono-colonized C3H/HeN (*N* = 8) and C57BL/6 (*N* = 3) mice. **(C)** Flow cytometry analysis of FoxP3^+^ Treg cells in the lamina propria of conventional mice treated for 14 days with *R. hominis*: C3H/HeN mice (*P = 0.04 between control and *R. hominis* treatment) and C57Bl/6 mice (**P = 0.05 between control and *R. hominis* treatment). **(D)** Flow cytometry analysis of FoxP3^+^ Tregs in lamina propria of FoxP3-tagged conventional mice treated for 8 days with *R. hominis*, with 4 days of low dose DSS treatment or untreated. Statistical significance: *P < 0.005 control versus DSS treatment and **P = 0.02 DSS treatment versus DSS^+^ *R. hominis* treatment. **(E)** Flow cytometry plots of FoxP3^+^ Treg cells in the colonic lamina propria of DSS-treated mice and DSS^+^ *R. hominis*-treated mice. Plots are representative of three experiments. The gating strategy for the FACS plots was as follows: FSC/SSC (removal of cell debris), live/dead-SSC, singlets (FSC-A/FSC-H), CD3^+^CD8^−^, and CD25^+^FoxP3^+^. The percentages were calculated as CD25^+^FoxP3^+^ cells of total CD4^+^ T cells in each preparation. CD4^+^ cells were derived from the gating strategy of CD3^+^CD8^−^.

Immune response was a major pathway induced by *R. hominis* at day 28 in the ascending colon of mono-associated mice. The pathways significantly affected in this category were mostly involved in T cell function, including IL-10 signaling and regulation of T cell function by CTLA-4 (Table S3 in Supplementary Material). The Ly6 gene family was also induced in the ascending colon. In particular, the GPI-anchored gene product of *Ly6g6c* was upregulated 25-fold, and the related gene *Ly6g6e* was upregulated twofold at day 28. The majority of hematopoietic cells, including neutrophils and plasmacytoid dendritic cells, express one or more members of the Ly6 family. The increased presence of Ly6G^+^ cells in *R. hominis*-colonized mice was confirmed by immunocytochemistry (Figure 3A).

While the pathways of T cell regulation were significantly influenced by association with *R. hominis*, the corresponding effects on T cell differentiation required further investigation. We therefore assessed the number of double-positive CD3^+^FoxP3^+^ cells in the colonic lamina propria of mono-associated C3H/HeN mice. A significant increase in the number of regulatory T cells (CD3^+^FoxP3^+^ cells) was detected in the germfree C3H/HeN and C56BL/6 mice colonized with *R. hominis* (Figures 3A,B).

Conventional C3H/HeN and C57BL/6 mice were gavaged with *R. hominis* for 14 days to determine the impact of the bacterium on Treg cells in the *lamina propria* of animals with a normal microbiota. This treatment resulted in a small but significant increase in the population of CD3^+^CD4^+^CD25^+^FoxP3^+^ T cells in the C3H/HeN (*P* = 0.04) and a smaller increase in the C57BL/6 mice (*P* = 0.05) when compared to non-colonized mice (Figure 3C). While the population of Tregs was induced in conventional mice after colonization with *R. hominis* under homeostatic conditions, it was unknown if this induction could be maintained under conditions of mild colitis. We therefore investigated the impact of the bacterium on Treg cells in the lamina propria of animals that were given low dose DSS. FoxP3-tagged mice of C57BL/6 background were colonized with *R. hominis* for 2 days before treatment with low dose of DSS for 4 days followed by 2 days of pure drinking water. DSS treatment alone significantly increased the population of CD3^+^CD4^+^CD25^+^FoxP3^+^ T cells (*P* = 0.02) (Figure 3D). When the animals were additionally treated with *R. hominis*, there was a further significant increase in the population of CD3^+^CD4^+^CD25^+^FoxP3^+^ T cells (*P* = 0.02) versus DSS (Figures 3D,E).

### The Role of *R. hominis* Flagellins in Modulation of T Cell Differentiation

**Figure 4:**
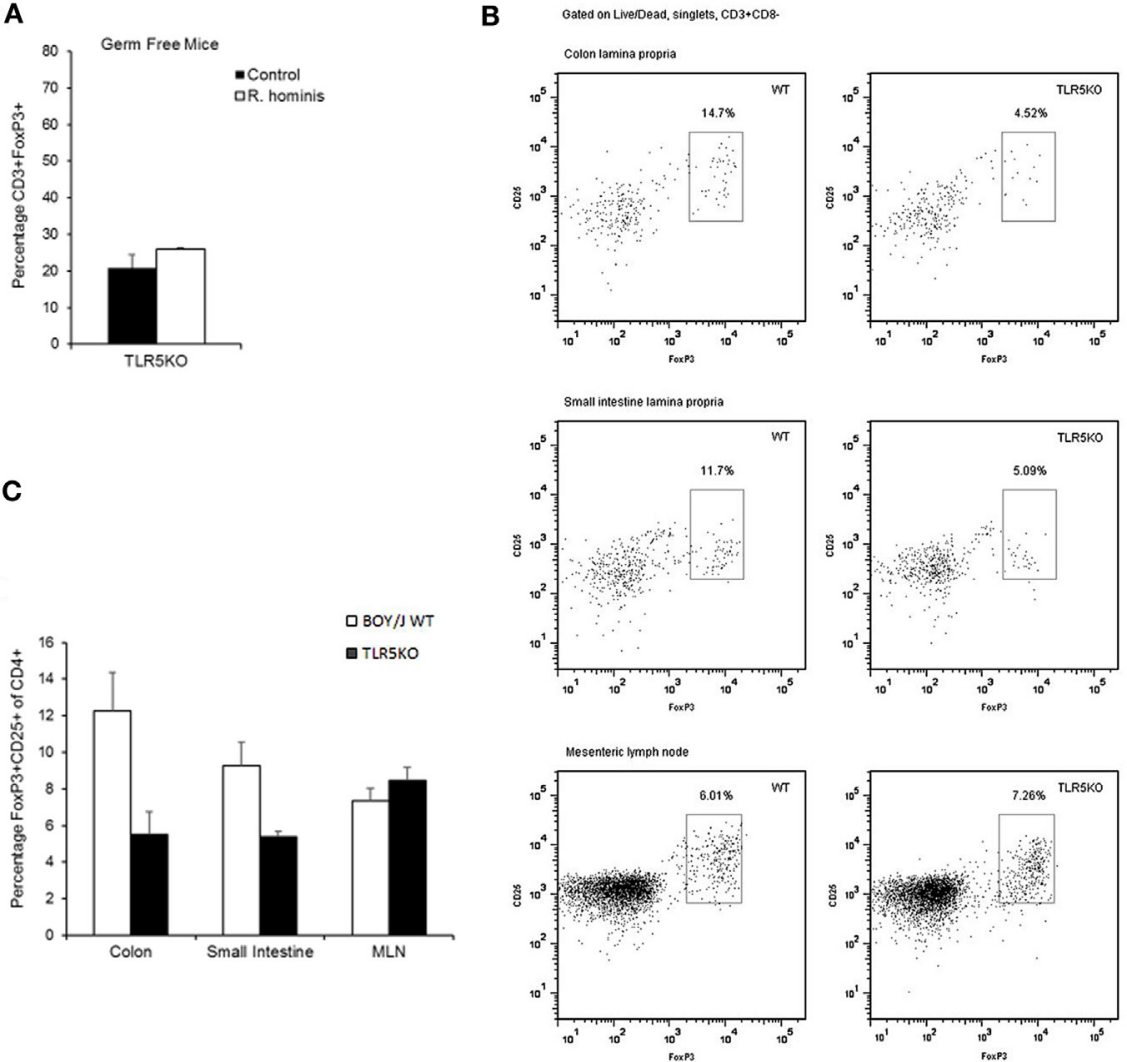
Modulation of T cell differentiation by *Roseburia hominis* flagellins. **(A)** Quantification of lamina propria cells in the ascending colon immunofluorescently labeled with anti-CD3 and anti-FoxP3 in germ-free (GF) and *R. hominis* mono-colonized TLR5KO mice (*N* = 2). **(B)** Flow cytometry plots of FoxP3^+^ Treg cells in the colonic and small intestinal lamina propria and mesenteric lymph nodes (MLNs) of conventional TLR5KO mice and Boy/J WT mice treated for 14 days with *R. hominis*. **(C)** Flow cytometry analysis of FoxP3^+^ Treg cells in conventional TLR5KO mice and Boy/J WT mice treated for 14 days with *R. hominis*: in the colonic lamina propria (*P* = 0.01 between TLR5KO and WT), in the lamina propria of the small intestine (*P* = 0.03 between TLR5KO and WT), and in MLNs (*P* = 0.33 between TLR5KO and WT).

The influence of bacteria on the differentiation of T cells may reflect a specific array of TLR ligands displayed by a particular bacterium. TLR5KO (GF and conventional) and WT murine strains were gavaged with the bacterium to evaluate the functional importance of *R. hominis* and its flagellins. Analysis of the differentially expressed genes in TLR5KO and WT Boy/J mice colonized with *R. hominis* revealed that although T cell pathways were still influenced by the colonization event in TLR5KO mice, responses were more related to IL-4, IL-5, IL-6, IL-9 pathways and not to IL-10 and CTLA-4 (Table S4 in Supplementary Material). Furthermore, in contrast to mono-associated C3H/HeN and C57BL/6 animals (Figure 3B) and conventional Boy/J animals (Figures 4B,C), the numbers of double-positive CD3^+^FoxP3^+^ cells in the *lamina propria* of TLR5KO mice were not increased due to *R. hominis* treatment (Figure 4A).

**Figure 5:**
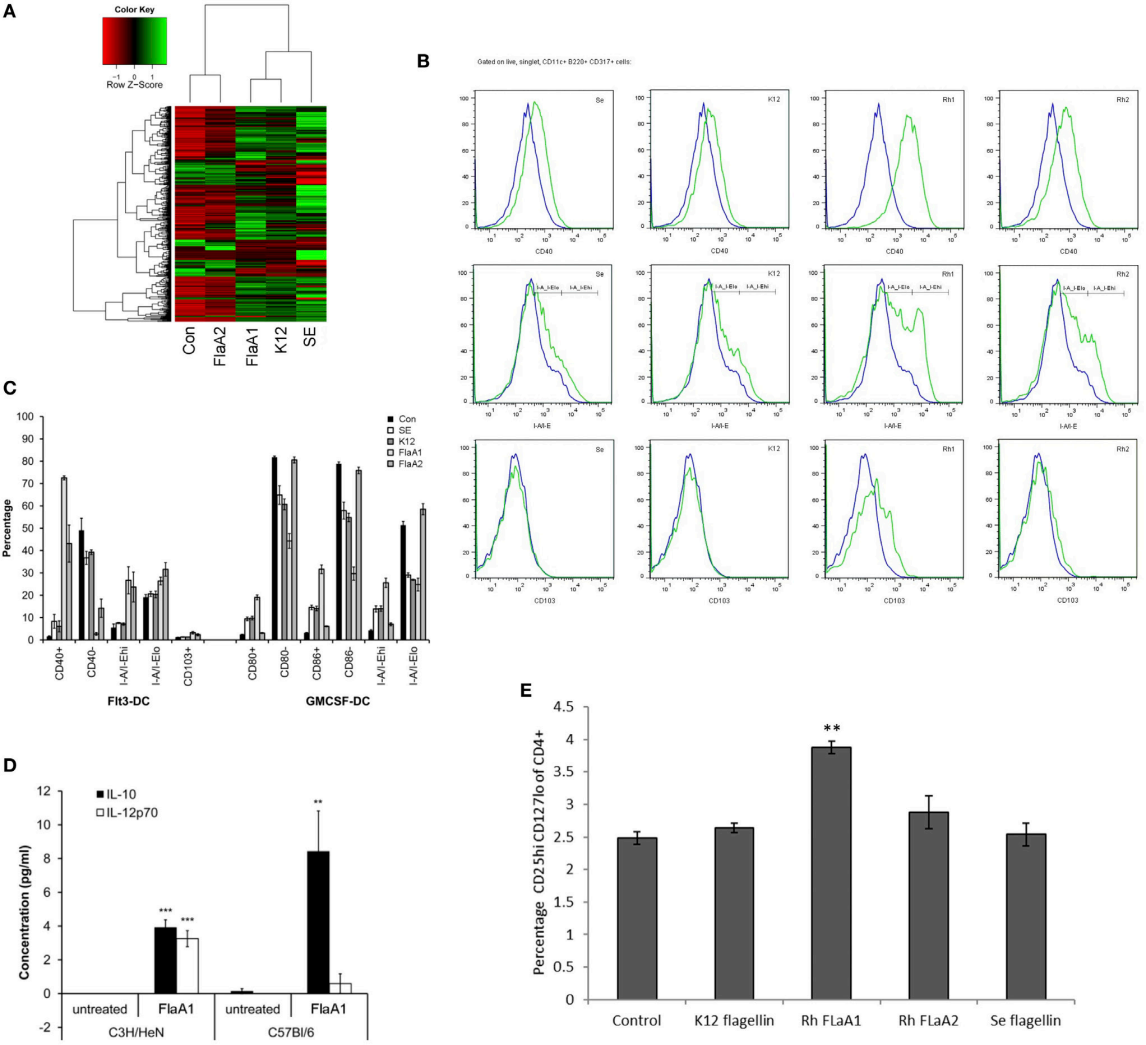
Effects of *Roseburia hominis* flagellin FlaA1 on intestinal epithelial cells and murine bone marrow-derived dendritic cells. **(A)** Heatmap of differentially expressed genes in Caco-2 cells treated with different bacterial flagellins: from *Salmonella enterica* serotype Enteritidis (SE), *Escherichia coli* K12 (K12), and *R. hominis* (FlaA1 and FlaA2). **(B)** Expression of CD40; I-A/I-E and CD103 by CD11c^+^B220^+^CD317^+^ Flt3L-derived dendritic cells from conventional C3H/HeN, control (blue) and after 24 h incubation with recombinant flagellins (SE, K12, FlaA1, FlaA2; all in green) determined by flow cytometry. Histogram represents data from three experiments. **(C)** Frequencies of Flt3L- and GM-CSF-derived dendritic cells from conventional C3H/HeN gated on CD11c^+^B220^+^CD317^+^ cells and CD11c^+^CD11b^+^B220^−^ cells treated with different recombinant flagellins (SE, K12, FlaA1, and FlaA2). Results are shown as the percentage of the total, live and singlet cells, mean ± SD from three experiments. **(D)** The level of IL-10 and IL-12 cytokines was measured by cytometry bead arrays in supernatants from control (unstimulated DCs; *N* = 3) and FlaA1-treated DCs (*N* = 3) derived from C3H/HeN and C57BL/6. Data are presented as mean ± SD. Statistical significance: ***P < 0.001. **(E)** Flagellin stimulated Flt3-derived dendritic cells were cocultured with CD4^+^ T cells for 5 days, and T cell were stained for the expression of T cell differentiation markers. Graph indicates the percentage of activated Treg cells. Statistical significance: **P < 0.01.

A set of flagellins from pathogenic and commensal bacteria, including recombinant *R. hominis* flagellins, was used to compare their effect on activation of signaling responses in IECs and BMDCs. Caco-2 cells were treated with the identical concentrations of different bacterial flagellins (Figure 5A). The flagellin of pathogenic *S. enterica* serotype Enteritidis (SE) induced a larger gene panel than the flagellins of commensal *E. coli* K12 or *R. hominis* FlaA1. *E. coli* and *R. hominis* induced a strong response and, with a similar gene complement between the two, formed a separate clade distinct from SE. In contrast, FlaA2 was generally neither pro-inflammatory nor did it activate the conserved gene signature (*IL8, CXCL1, CXCL2*, and *CXCL10*) that was induced by the other recombinant bacterial flagellins. In fact, the set of genes affected was closer to that of the control cells and formed a cluster separated from the other flagellins. FlaA1 from *R. hominis* induced different responses in Flt3L- and GM-CSF-derived BMDCs compared to SE and K12 (Figures 5B,C). In particular, FlaA1 was uniquely able to activate Flt3L-expanded DCs, with the upregulation of I-A/I-E and CD40 and production of IL-10 by BMDCs from both C3H/HeN and C57BL/6 mice. The IL-10/IL-12 ratio was particularly elevated in C57BL/6 DCs (Figure 5D), which were found to be CD103^+^Siglec-H^+^. To test whether flagellin activated BMDCs could differentially modulate naïve T cell differentiation, mixed leukocyte cultures of CD4^+^ OTII cells with flagellin stimulated Flt3-derived BMDC were performed. The data show that the production of IL-10 and IL-12 by the flagellin stimulated Flt3-derived BMDC ratio triggered naïve T cell differentiation toward a Tregs profile (Figure 5E). In contrast to FlaA2, FlaA1 significantly increased the Treg population in the T cell cocultures.

### *R. hominis* Attenuates Colitis in DSS-Treated Mice

**Figure 6:**
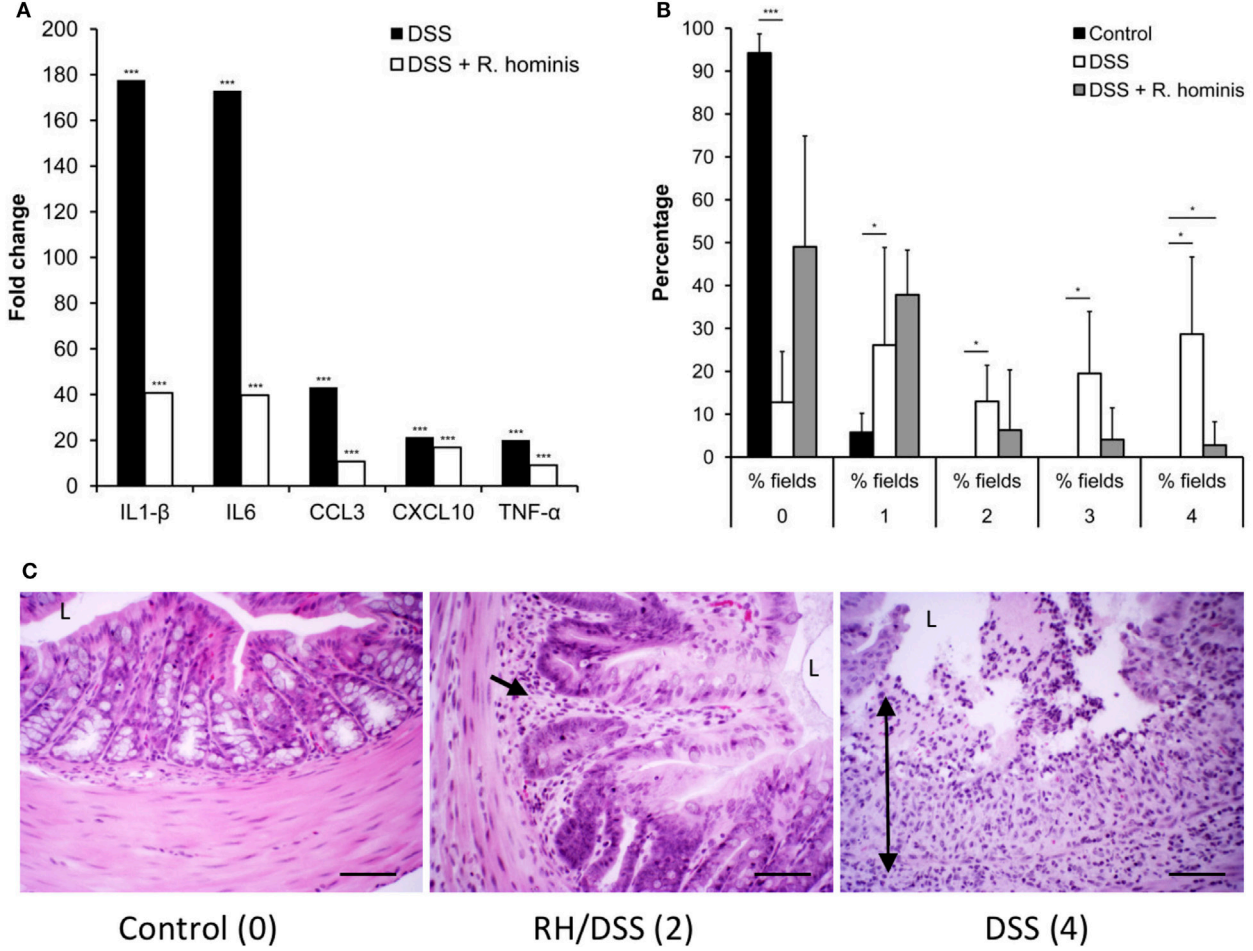
*Roseburia hominis* attenuates inflammation in the DSS model of colitis. **(A)** DSS mice untreated with *R. hominis* (*N* = 8) had strong elevation of all genes compared to the control mice (*N* = 4), while differential gene expression was lower in *R. hominis*-treated animals (*N* = 10). RT-qPCR results obtained on day 15 are presented as a fold change compared to control. Statistical significance: ***P < 0.001. **(B)** Histopathology tissue scoring (25) presented as mean percentage of fields of view at a given grade. DSS treatment significantly altered all fields of view at grades 0, 2, 3, and 4. *R. hominis* significantly reduced the % fields of view for grade 4 pathology (*P* = 0.02) and increased the % fields of view for grade 0. Data obtained on day 15 are presented as mean ± SD. **(C)** Ascending colon (hematoxylin/eosin stained) of control, DSS^+^ *R. hominis*-treated and DSS-treated animals. Images shown are the representative fields of view for each treatment group with the allocated score in parenthesis. Single black arrow head indicates infiltrating cells, and the double headed arrow indicates transmural infiltration. L, lumen. Original magnification ×200. Scale bar represents 50 µm.

The effects of *R. hominis* on innate and adaptive immunity prompted us to test its therapeutic efficacy using the murine DSS model (Figure S2 in Supplementary Material; Figure 6). The treatment group was dosed daily with *R. hominis* (~50 μL, ca. 10^9^ CFU) *via* gavage for a period of 14 days while the untreated group received the same amount of bacterial growth medium. The two groups were then given DSS in drinking water (MW 50 kDa, 30 g/L) beginning from day 8 onward. On day 15, DSS-treated mice without *R. hominis* addition had a strong elevation of a panel of pro-inflammatory biomarkers compared to control mice, with gene induction levels ranging from 4- to 49-fold (Figure 6A). Induction of pro-inflammatory genes was significantly lower in the *R. hominis*-treated mice compared to the control group, indicating a strong therapeutic benefit of oral administration of *R. hominis*. On day 15, severe inflammation was detected in the ascending colon of DSS-treated mice without an *R. hominis* supplement, while the colonic mucosa in the *R. hominis*-supplemented group displayed only low-level inflammation, consistent with the reduced expression of pro-inflammatory genes (Figures 6B,C). Treatment with *R. hominis* also alleviated the weight loss in DSS-treated mice (Figure S3 and Table S5 in Supplementary Material).

### *R. hominis* Colonization Influences Body Composition and Expression of Satiety Genes

In the next set of experiments, we investigated metabolic changes in the host and bacterium due to host–microbe association. Mice mono-associated with *R. hominis* displayed significant metabolic changes compared to control GF mice. In particular, the GO processes “negative regulation of response to food” (GO:0032096), “negative regulation of appetite” (GO:0032099), and “regulation of catecholamine secretion” (GO:0050433) were all downregulated in the ascending colon after colonization by *R. hominis* [NCBI GEO (Gene Expression Omnibus), accession number GSE25544]. The genes involved in these processes were *Agt, Cartpt, Cck*, and *Cxcl12*, with the corresponding mRNA changes induced ranging from 2- to 12-fold. *Cck*, in particular, plays a major role in digestion and satiety as a hunger suppressant. *Gcg* expression was also downregulated at this gut site.

**Figure 7:**
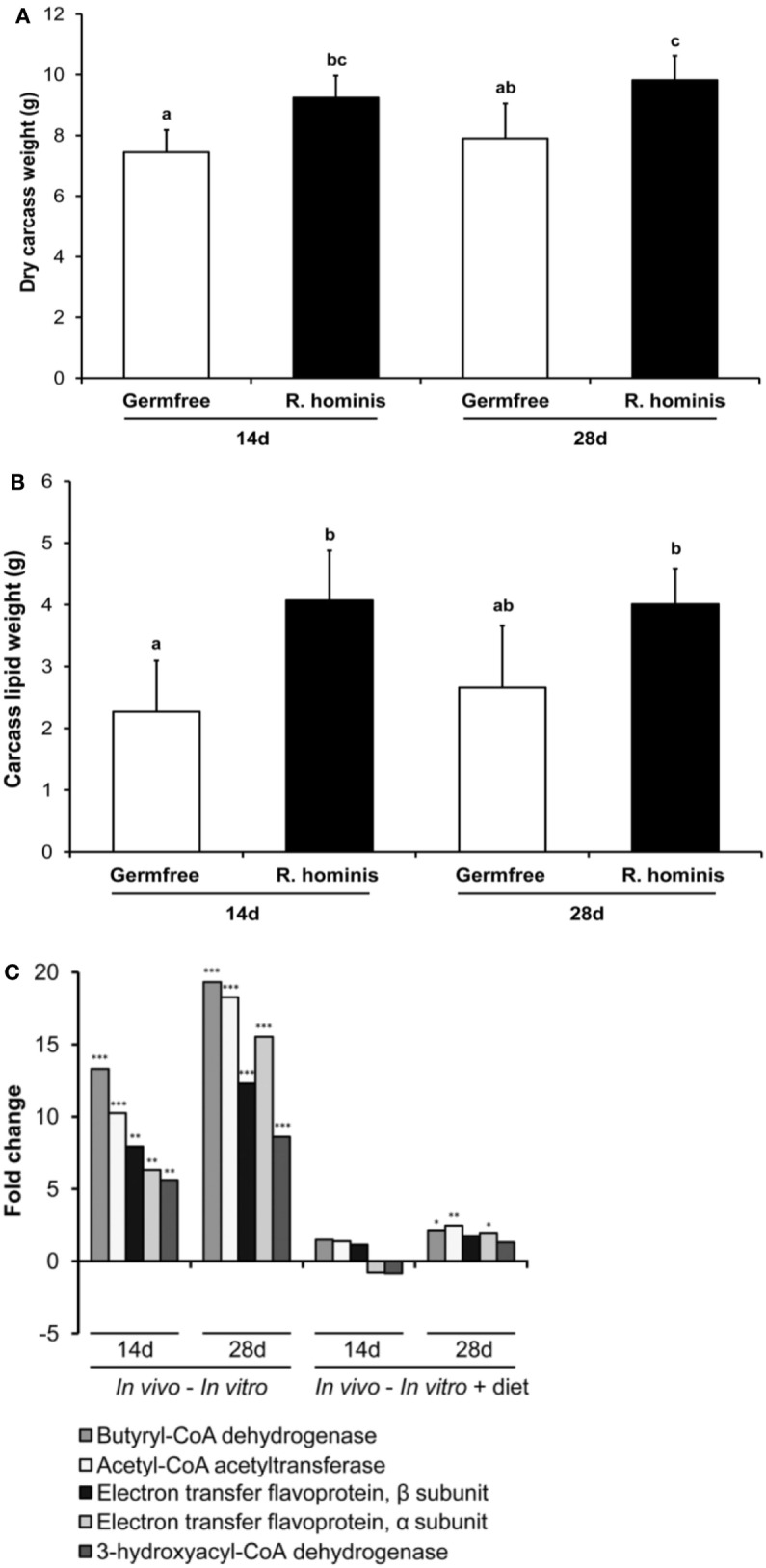
*Roseburia hominis* colonization influences satiety genes and body weight. The germ-free (GF) C3H/HeN male mice were allocated into the control (*N* = 8) and treatment (*N* = 10) groups. At days 0, 1, and 2, animals in the treatment group were dosed by ~10^9^ CFU of *R*. *hominis* in YCFA medium (100 µL) by gavage, while control animals were given 100 µL of YCFA medium alone. Dry body weight and lipid content of carcasses were analyzed. **(A)** Weight of dry carcasses of *R. hominis*-associated mice was significantly heavier compared to GF animals. **(B)** Carcass lipid analyses showed that the total adiposity was also significantly higher in *R. hominis*-associated animals at day 14. Data are presented as mean ± SD. **(C)** RT-qPCR analysis of genes involved in butyrate metabolism.

To establish whether the transcriptional changes of these genes have any physiological relevance in terms of body composition, analyses of dry carcass weight and lipid composition were performed. Dry carcass weights of *R. hominis*-associated mice were significantly heavier than those of GF animals (Figure 7A). Carcass lipid analysis showed that the total adiposity was also significantly higher in *R. hominis*-colonized animals at day 14 (Figure 7B).

Significant metabolic changes were also detected on the other side of the host–microbe interaction spectrum. In particular, expression of *R. hominis* genes involved in butyrate production was upregulated by the gut environment (Figure 7C). These were the genes encoding acetyl-CoA acetyltransferase, 3-hydroxyacyl-CoA dehydrogenase, butyryl-CoA dehydrogenase, and phosphoenolpyruvate carboxykinase. The stimulatory effect of host epithelial cells on production of acetate and butyrate by *R. hominis* was further confirmed in separate *in vitro* experiments (Figure S4 in Supplementary Material). Bacterial cells incubated with Caco-2 and HT29 cells produced significantly higher amounts of these metabolites compared to *R. hominis* control cells incubated under the same conditions but without the epithelial cells.

## Discussion

A long-term co-evolution of host–microbe interaction has likely driven the selection of functionally important bacterial species in the gut, the majority of which are not encountered in other ecosystems. There is only limited information available at present about the contribution of individual members of the microbial community to intestinal functions, in particular to the development and regulation of mucosal immunity.

The specific functions of certain intestinal bacteria such as *B. fragilis* and SFB have been investigated in the mouse gut to define their individual contributions to T cell biology, and these bacteria have been shown to be potent inducers of Tregs and Th17 cells (4, 7, 8). Members of the *Clostridium* clusters IV and XIVa have recently received more attention. For instance, their presence in the altered Schaedler flora and the contribution of a mixed culture of *Clostridium* spp. to T cell differentiation has also been noted (9, 10).

We report here the successful and stable mono-association of *R. hominis*, a strictly anaerobic flagellated member of the Firmicutes phylum, in the gut of GF mice. The transcriptional responses of *R. hominis* following mono-colonization could be attributed to both the host gut environment and diet. These included chemotaxis and motility subsystems. The role of motility and the flagellar apparatus in host colonization is well understood for pathogenic bacteria but less is known about the role of flagellins in commensal bacteria. Genes encoding cell motility functions, and flagellins in particular, are generally expressed at variable and low abundance rates in the gut microbiome (27). In addition, only a subset of human gut microbiomes contains detectable flagellin genes (28). We showed that *R. hominis* flagellins are expressed in the mouse intestinal environment. Flagellin gene expression appears to be partly dependent on the presence of certain dietary constituents. Previous work has shown that flagellin gene expression of the related species *Roseburia inulinivorans* is indeed substrate dependent; its expression is higher in the presence of starch compared to glucose, inulin, or fructan (29).

The presence of *R. hominis* in the gut induces genes involved in promoting gut barrier function and innate immunity. Tight junctions, gap junctions, and adherens junctions operate to limit bacterial translocation to the subepithelial layer of the gut, and these functions may be promoted by intestinal bacteria (30). Both CD and UC are characterized by the loss of barrier function and the integrity of tight junctions. Interestingly, dysbiosis of the gut microbiota in UC and CD is associated with a significant reduction of *R. hominis* and *F. prausnitzii* (16, 19). Our results concerning the active contribution of *R. hominis* to gut barrier function support the view that its loss in patients with CD or UC may have significant consequences. Tight junction complexes can be activated by a number of other commensal and probiotic bacteria (31), potentially contributing to the amelioration of the “leaky gut” condition in these diseases.

*Roseburia hominis* induced the expression of genes such as *Ly6g6c*, as well as pathways involved in the regulation of T cells. The most affected T cell pathways included those related to IL-10, ICOS, and CTLA-4, which are all involved in the differentiation of Treg cells. Significant increases in colonic CD3^+^CD4^+^CD25^+^FoxP3^+^ cells were observed in mice either mono-colonized with *R. hominis* or in conventional mice fed a supplement of live *R. hominis* culture. Our findings complement recent reports on Treg differentiation effects by other *Clostridium* species (10, 32). We have shown here that the single bacterial strain *R. hominis* A2-183 is able to promote mucosal T cell expansion and impact T cell differentiation in mono-associated, conventional and DSS-treated mice.

Members of the *Roseburia* genus are some of the most prevalent motile bacterial species in the normal healthy human intestinal microbiota (33). Flagellins are potent immunomodulatory proteins: flagellated bacteria, such as *R. hominis*, could interact actively with the host immune system. Another important consequence of motility among the *Roseburia* species is their ability to penetrate the mucus layer and attach to the host gut epithelial cell surfaces (34). This property is considered to be a very desirable characteristic enhancing the probiotic potential of bacteria (35). Close proximity to the host cells allows commensal gut bacteria to exert potent physiological effects, potentially reversing metabolic disorders and controlling inflammation, gut barrier function, and gut peptide secretion.

Flagellin signaling by many pathogenic bacteria through the host’s TLR5 receptors induces a strong pro-inflammatory response, mainly driven by the activation of the NF-κB signaling pathway (36). However, the numerical prevalence of flagellated commensal bacteria makes a strong pro-inflammatory scenario seem unlikely. These bacteria are capable of flagellin/TLR5 signaling, which is important for host defense and disease protection, because deletion of TLR5 results in colitis (37). The presence of flagellin genes in the SFB genomes (38, 39) may explain the potent induction of Th17 cells by these bacteria (7, 8).

Differential expression of *R. hominis* flagellins *in vitro* has effects on IECs and BMDCs. The panel of flagellins tested affected IECs and BMDCs differently, although all the flagellin structures included the conserved Arg90 domain associated with flagellins that bind and activate TLR5 (40), which suggests that other sequence/structural properties might account for the signaling responses mediated by FlaA1 and FlaA2. Certain commensal flagellin structures may help to direct immune tolerance responses through TLR5 expressed on either CD103^+^DC or Treg subsets (23, 41, 42). The significance of flagellin-TLR5 signaling in Treg responses induced by *R. hominis* was further investigated using TLR5KO mice. We showed that *R. hominis*-induced CD3^+^CD4^+^CD25^+^FoxP3^+^ cells were significantly lower in TLR5KO mice, indicating that TLR5/flagellin signaling is an important mediator for the expansion of this T cell subset. The immunomodulatory effects of *R. hominis* were shown in DSS-treated mice. Although the DSS model of colitis is generally a T cell-independent model, we showed that *R. hominis* induced regulatory T cell populations in these animals. Other anti-inflammatory molecules such as butyrate may also contribute to immune tolerance and limit tissue damage (43, 44). Another effect of short-chain fatty acids produced by the bacterium is the improved food conversion efficiency. The dietary constituents such as polysaccharides that are poorly utilized by the host are degraded by the bacterium and used by the host resulting in a better energy recovery from the diet. Differential expression of satiety genes may be another factor contributing to the heavier weight and higher adiposity in *R. hominis*-colonized animals.

In summary, gut colonization with *R. hominis* caused the induction of specific subsets of genes on both the bacterial and host sides of the interaction. In this study, we have focused on signaling amongst other molecular mechanisms to explain the nature of this cross talk. TLR5/flagellin signaling could, potentially, drive the expansion of Treg cells *via* a TLR5-dependent mechanism, and our results suggest a potential therapeutic benefit of *R. hominis* in the treatment of UC, which is characterized by the loss of this bacterium from the gut (16).

## Ethics Statement

The management and experimental procedures with animals were approved by the respective Local Ethical Review Committees at Institut Micalis, INRA, Jouy-en-Josas, France and Medical Research Facility, University of Aberdeen, United Kingdom.

## Author Contributions

AP, IM, and RA designed research; AP, IM, AT, AL, NC-B, VG-R, KG, EL, MD, AC, EM, VF, RI, GG, and RA performed research; AP, IM, AL, GG, and RA analyzed data; AP, IM, GG, and RA wrote the manuscript.

## Conflict of Interest Statement

The authors declare that the research was conducted in the absence of any commercial or financial relationships that could be construed as a potential conflict of interest.

## Footnotes

^1^http://www.r-project.org.

^2^http://www.bioconductor.org.
