# Supplementary material for: Human Gut Symbiont Roseburia hominis Promotes and Regulates Innate Immunity
Source: Front Immunol. 2017 Sep 26;8:1166. doi: 10.3389/fimmu.2017.01166 (PMC5622956; doi:10.3389/fimmu.2017.01166)
Supplement: Supplementary file 1 [file Image_1.PDF]

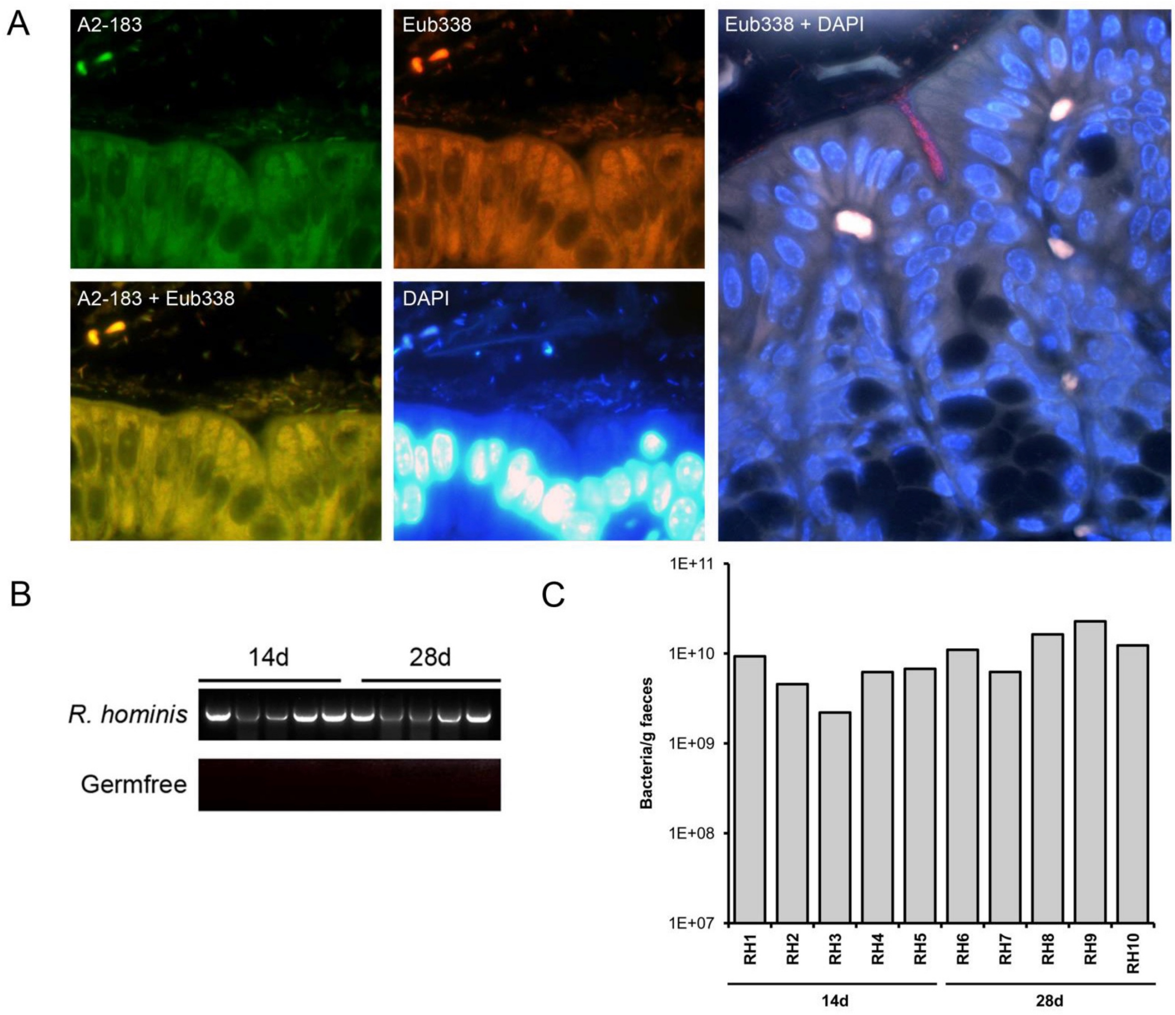

Fig. S1. *R. hominis* preferentially colonizes the ascending colon of GF mice. A - *R. hominis* colonizes the ascending colon of mice, with close association of bacteria to the host epithelium, which is detected by FISH using A2-183 (*R. hominis* A2-183-specific probe; FITC), Eub338 (bacterial universal probe; Cy3) and DAPI (nuclei; blue). Composite images of A2-183+Eub338 and Eub338+DAPI are also shown. The gamma for the red channel was increased in the Eub338+DAPI composite to illustrate the labeled bacteria. Original magnification is x630. B - PCR using *R. hominis*-specific primers showed a strong positive signal in fecal DNA post-colonization, while feces of GF animals were negative for the presence of any bacteria. C - qPCR analysis showing colonization levels of *R. hominis*/g feces. Bacterial DNA isolated from feces was compared against known standard concentrations of *R. hominis* grown in culture. The similar bacterial levels were detected in all mono-colonized mice, with approximately $1x10^{10}$ bacteria/g feces. Feces of GF animals were tested negative for the presence of any bacteria.
