# Supplementary material for: Human Gut Symbiont Roseburia hominis Promotes and Regulates Innate Immunity
Source: Front Immunol. 2017 Sep 26;8:1166. doi: 10.3389/fimmu.2017.01166 (PMC5622956; doi:10.3389/fimmu.2017.01166)
Supplement: Supplementary file 2 [file Image_2.PDF]

A

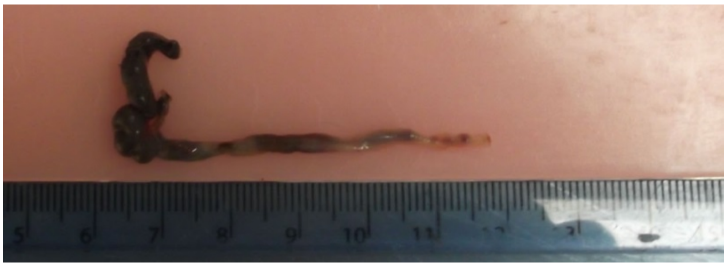

B

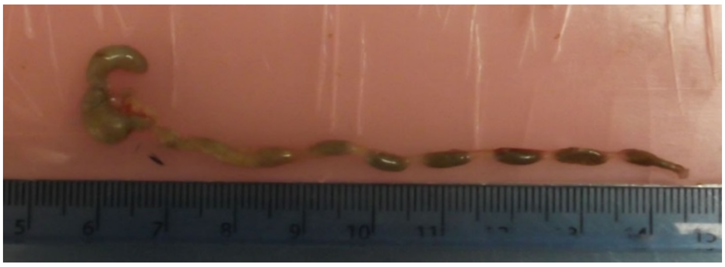

C

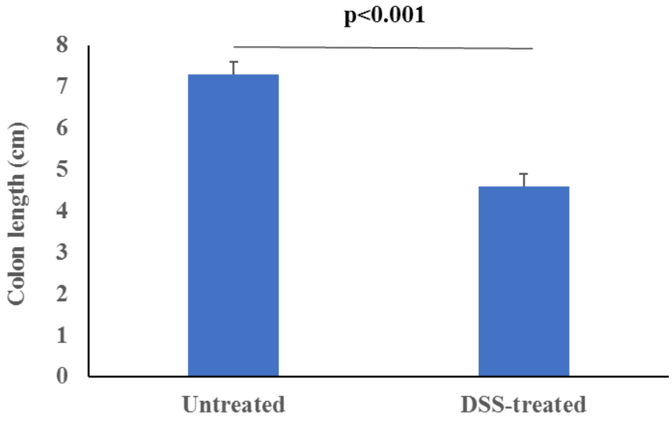

Figure S2. Representative photographs of the caecum and colon of mice in the DSS model of colitis. A - mice given drinking water containing DSS (30g/l). B – mice given pure drinking water. C – Average colon length in DSS-treated and control groups.
