# Supplementary material for: Human Gut Symbiont Roseburia hominis Promotes and Regulates Innate Immunity
Source: Front Immunol. 2017 Sep 26;8:1166. doi: 10.3389/fimmu.2017.01166 (PMC5622956; doi:10.3389/fimmu.2017.01166)
Supplement: Supplementary file 3 [file Image_3.PDF]

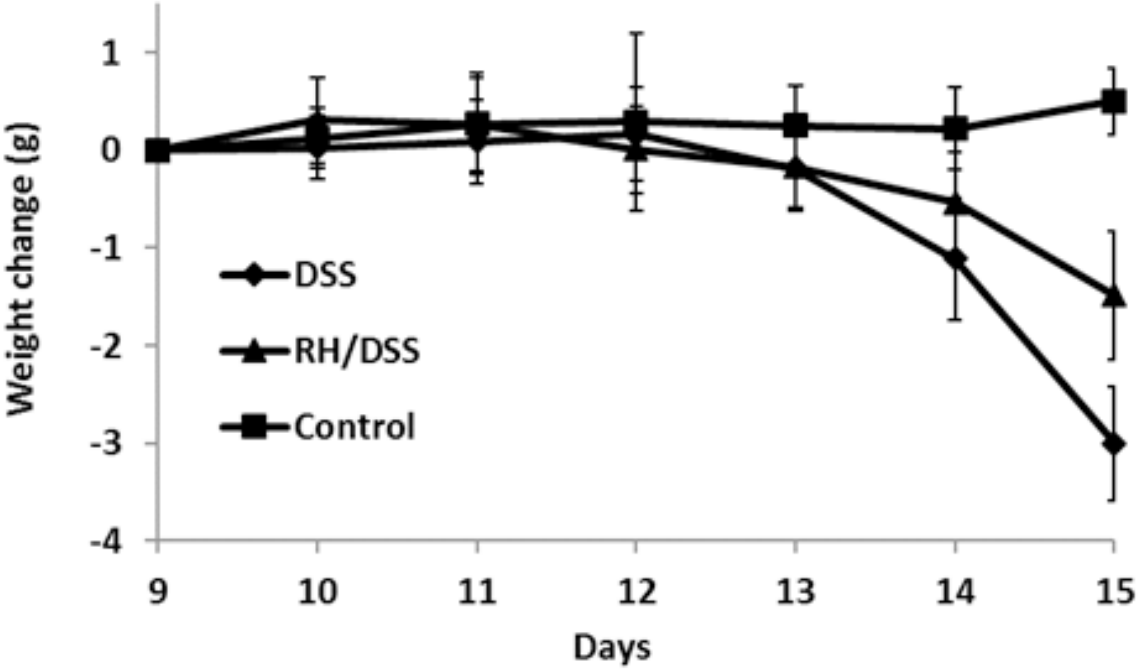

Fig. S2. *R. hominis* limits body weight loss in the DSS model of colitis. Mice consuming DSS (N=18) had a rapid decline in body weight from day 12 (after 4 days intake of DSS) compared to the control mice (N=8) and had lost 16±3 percent of their body weight when euthanized on day 15 (after 6 days of DSS). Loss of weight was also evident in mice given DSS and *R. hominis* (N=18) but it was less marked than with DSS alone (-8±4 percent of body weight by day 15; p<0.001 versus DSS alone). All mice used were female C57BL/6.
