# Supplementary material for: Human Gut Symbiont Roseburia hominis Promotes and Regulates Innate Immunity
Source: Front Immunol. 2017 Sep 26;8:1166. doi: 10.3389/fimmu.2017.01166 (PMC5622956; doi:10.3389/fimmu.2017.01166)
Supplement: Supplementary file 4 [file Image_4.PDF]

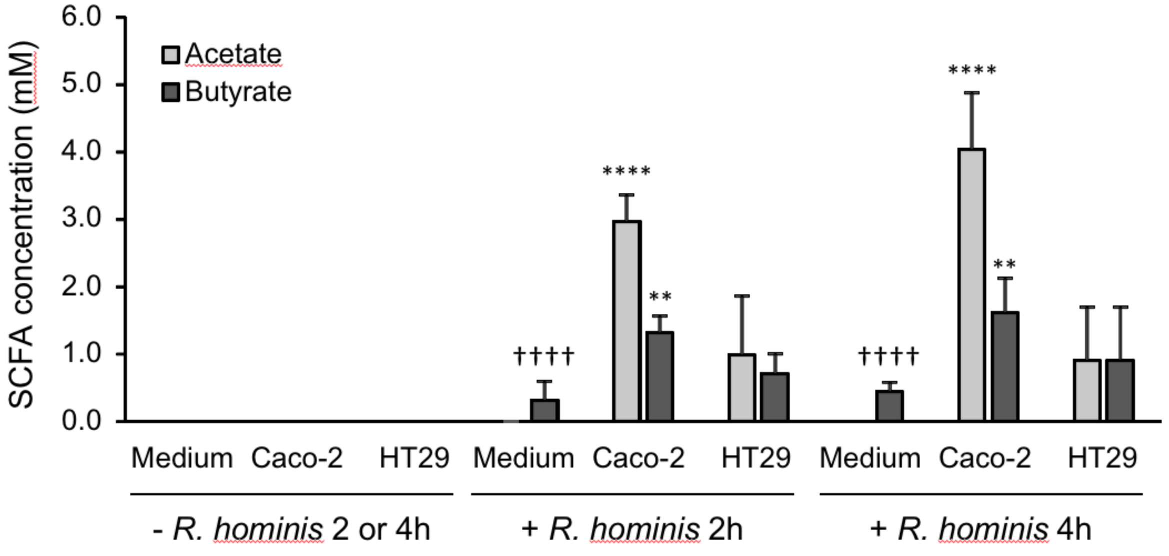

Fig. S4. Stimulatory effect of Caco-2 and HT29 cells on acetate and butyrate production by *R. hominis*. Confluent Caco-2 and HT29 cells were incubated in the presence (+) or absence (-) of *R. hominis* for 2 and 4h in cell apical anaerobic culture medium containing glucose (DMEM). Medium - incubation of *R. hominis* alone in DMEM. Acetate and butyrate concentrations are expressed as mean ± SD (n = 3/group). Production of acetate and butyrate by *R. hominis* is stimulated by the presence of epithelial cells ($p < 0.05$). †††† - $p < 0.0001$ compared to the medium without the bacterium; ** - $p < 0.01$ and **** - p < 0.0001 compared to *R. hominis* incubated in the medium alone.
