# Supplementary material for: Human Gut Symbiont Roseburia hominis Promotes and Regulates Innate Immunity
Source: Front Immunol. 2017 Sep 26;8:1166. doi: 10.3389/fimmu.2017.01166 (PMC5622956; doi:10.3389/fimmu.2017.01166)
Supplement: Supplementary file 5 [file Table_1.PDF]

Table S1. Index of RT-qPCR experiments positioned on the genomic map of *R. hominis* (GenBank accession number CP003040).

Exp	Primer	Start	End	Length	Coding sequence
1	gyrA-N-F	7803	7889	87 bp	GyrA
2	1602-F	95664	95740	77 bp	Phosphate regulon sensor protein PhoR
3	1653-F	153403	153483	81 bp	Flagellin protein FlaA1
4	1686-F	189295	189382	88 bp	Flagellin protein FlaA2
5	1718-F	221205	221279	75 bp	Flagellin protein FlaA3
6	1735-F	250582	250674	93 bp	Methyl-accepting chemotaxis protein1
7	1769-F	290546	290628	83 bp	Methyl-accepting chemotaxis sensory transducer1
8	1770-F	291722	291808	87 bp	Methyl-accepting chemotaxis protein2
9	1831-N-F	348711	348810	100 bp	MobA/MobL protein4
10	1842-F	364775	364851	77 bp	MobA/MobL protein2
11	1867-2652-F	391044	391120	77 bp	MobA/MobL family protein4/putative conjugal transfer protein
12	2055-F	600837	600928	92 bp	Acetyl-CoA acetyltransferase
13	2056-F	602279	602363	85 bp	3-hydroxyacyl-CoA dehydrogenase
14	2057-F	602961	603037	77 bp	Butyryl-CoA dehydrogenase
15	2058-F	604411	604504	94 bp	Electron transfer flavoprotein, beta subunit
16	2059-F	605434	605516	83 bp	Electron transfer flavoprotein, alpha subunit
17	129-F	653987	654066	80 bp	Oligopeptide ABC transporter, periplasmic oligopeptide-binding protein oppA
18	132-F	658435	658516	82 bp	Oligopeptide transport ATP-binding protein oppD
19	805-R	934310	934406	97 bp	Osmosensitive K+ channel histidine kinase KdpD
20	807-R	935306	935394	89 bp	Aldose epimerase family protein
21	808-R	936111	936190	80 bp	Potassium uptake protein, integral membrane component, KtrB
22	909-F	1053529	1053604	76 bp	Pyruvate-flavodoxin oxidoreductase
23	1235-F	1434705	1434785	81 bp	MobA/MobL protein3
24	1296-F	1495460	1495544	85 bp	Methyl-accepting chemotaxis protein3
25	1297-R	1497854	1497931	78 bp	L-threonine 3-O-phosphate decarboxylase
26	1335-F	1540579	1540671	93 bp	Flagellar motor rotation protein MotA
27	1336-F	1541416	1541511	96 bp	Flagellar motor rotation protein MotB
28	1356-F	1559143	1559227	85 bp	RNA polymerase sigma factor for flagellar operon
29	3119-F	2211612	2211705	94 bp	Phosphoenolpyruvate carboxykinase [ATP]
30	3117-R	2213046	2213139	94 bp	Methyl-accepting chemotaxis sensory transducer2
31	1867-2652-R	2736100	2736176	77 bp	MobA/MobL family protein4/putative conjugal transfer protein
32	1552M-R	2984489	2984566	78 bp	Magnesium transporter
33	397-R	3153341	3153427	87 bp	ATP synthase beta chain2
34	398-R	3153616	3153699	84 bp	ATP synthase gamma chain2
35	399-R	3155799	3155898	100 bp	ATP synthase alpha chain2
36	404-R	3159387	3159467	81 bp	Glucuronide permease
37	2399-R	3308172	3308265	94 bp	Putative conjugal transfer protein MobA/MobL
38	2323-R	3366526	3366615	90 bp	Flagellin protein flaB
39	2281-R	3416947	3417042	96 bp	ATP synthase beta chain
40	2280-R	3418736	3418824	89 bp	ATP synthase gamma chain
41	2279-R	3418857	3418942	86 bp	ATP synthase alpha chain
42	641-R	3467164	3467255	92 bp	MobA/MobL protein1
