# Supplementary material for: Human Gut Symbiont Roseburia hominis Promotes and Regulates Innate Immunity
Source: Front Immunol. 2017 Sep 26;8:1166. doi: 10.3389/fimmu.2017.01166 (PMC5622956; doi:10.3389/fimmu.2017.01166)
Supplement: Supplementary file 6 [file Table_2.PDF]

Table S2. RT-qPCR analysis of *R. hominis* mRNAs in response to gut environment.

Gene name	Rh14d - Control[a]		Rh28d - Control		Rh14d - *In Vitro*+diet		Rh28d - *In Vitro*+diet		*In Vitro* + diet – *In Vitro*	
	FC	*P*-value	FC	*P*-value	FC	*P*-value	FC	*P*-value	FC	*P*-value
3-hydroxyacyl-CoA dehydrogenase	5.62	0.00393	8.62	0.00000	-0.85	0.69927	1.30	0.38670	6.62	0.00124
Acetyl-CoA acetyltransferase	10.25	0.00304	18.27	0.00000	1.38	0.51556	2.46	0.01882	7.44	0.00048
Aldose epimerase family protein	7.20	0.00002	11.09	0.00001	22.65	0.03173	34.89	0.02105	-0.32	0.30489
ATP synthase alpha chain	1.62	0.12713	2.50	0.00003	3.25	0.00645	5.00	0.00000	-2.00	0.00147
ATP synthase alpha chain2	11.94	0.00122	5.50	0.00001	2.71	0.18580	1.25	0.72876	4.41	0.06479
ATP synthase beta chain	1.85	0.08341	3.14	0.00056	4.00	0.00397	6.80	0.00001	-2.16	0.00241
ATP synthase beta chain2	8.27	0.00058	6.13	0.00025	1.64	0.33264	1.21	0.67923	-5.05	0.01400
ATP synthase gamma chain	2.31	0.02994	3.58	0.00027	4.22	0.00238	6.52	0.00001	-1.82	0.01276
ATP synthase gamma chain2	-9.59	0.00081	-10.55	0.00013	-1.59	0.40064	-1.75	0.29398	-6.02	0.01363
Butyryl-CoA dehydrogenase	13.31	0.00166	19.32	0.00000	1.48	0.39908	2.14	0.02840	9.03	0.00039
Electron transfer flavoprotein, alpha subunit	6.32	0.00729	15.55	0.00000	-0.80	0.61916	1.96	0.03441	7.94	0.00030
Electron transfer flavoprotein, beta subunit	7.94	0.00414	12.31	0.00000	1.13	0.78504	1.76	0.08347	7.00	0.00078
Flagellar motor rotation protein MotA	-0.94	0.71937	2.37	0.00023	1.69	0.02442	4.25	0.00001	-0.56	1.00000
Flagellar motor rotation protein MotB	-0.57	0.01030	1.19	0.00938	-0.75	0.14601	1.58	0.01676	-0.76	0.08011
Flagellin protein FlaA1	-3.05	0.00125	-1.93	0.01193	3.13	0.01261	4.95	0.00249	-9.57	0.00079
Flagellin protein FlaA2	1.04	0.84732	-1.28	0.05814	1.45	0.29909	1.09	0.77089	-1.39	0.28105
Flagellin protein FlaA3	1.14	0.41467	1.94	0.00312	1.63	0.08606	2.77	0.00477	-1.43	0.17174
Flagellin protein flaB	1.02	0.97319	-4.99	0.00568	1.96	0.40946	-2.60	0.14359	-1.92	0.25258
Glucuronide permease	-9.34	0.00001	-13.81	0.00022	-13.97	0.00001	-20.65	0.00003	-1.50	0.13959
L-threonine 3-O-phosphate decarboxylase	1.62	0.00963	3.70	0.00002	1.66	0.07350	3.78	0.00186	-0.98	0.92518
Magnesium transporter	372.00	0.00123	11.20	0.03391	4.42	0.32048	-0.13	0.18758	84.10	0.01672
Methyl-accepting chemotaxis protein1	-1.18	0.54522	-1.83	0.02731	-2.90	0.01211	-1.87	0.05778	-0.29	0.00389
Methyl-accepting chemotaxis protein2	-2.46	0.00400	-2.95	0.00154	1.48	0.10070	1.24	0.33586	-3.65	0.00043
Methyl-accepting chemotaxis protein3	1.13	0.54189	1.86	0.04504	3.17	0.00033	5.25	0.00055	-2.81	0.00020
Methyl-accepting chemotaxis sensory transducer1	1.33	0.01743	2.15	0.00001	1.58	0.05704	2.56	0.00396	-1.19	0.38997
Methyl-accepting chemotaxis sensory transducer2	2.03	0.00671	2.34	0.00017	4.94	0.00007	5.71	0.00003	-2.44	0.00163
MobA/MobL family protein4/putative conjugal transfer protein	84.83	0.00177	5.77	0.01561	3.67	0.34227	-4.00	0.29305	23.10	0.04583
MobA/MobL protein1	257.93	0.00472	9.27	0.04437	8.72	0.25455	-3.19	0.50297	29.57	0.07733
MobA/MobL protein2	714.11	0.00172	11.64	0.08286	8.17	0.29225	-7.51	0.31871	87.43	0.04300
MobA/MobL protein3	362.26	0.00144	11.10	0.03880	7.77	0.27426	-4.20	0.43208	46.62	0.06260
MobA/MobL protein4	219.75	0.00147	6.52	0.08511	7.99	0.17452	-4.22	0.34388	27.49	0.04519
Oligopeptide ABC transporter, periplasmic oligopeptide-binding protein oppA	1.26	0.49437	1.11	0.45125	1.28	0.49171	1.13	0.53525	-1.02	0.92102
Oligopeptide transport ATP-binding protein oppD	-1.29	0.05256	1.08	0.40875	-1.40	0.07422	-1.00	0.98737	1.09	0.60319
Osmosensitive K+ channel histidine kinase KdpD	3.98	0.00004	7.07	0.00000	5.58	0.00005	9.91	0.00001	-0.71	0.12049
Phosphate regulon sensor protein PhoR	2.73	0.00211	1.54	0.04166	3.80	0.00919	2.14	0.07191	-1.39	0.34941
Phosphoenolpyruvate carboxykinase [ATP]	2.30	0.05000	3.22	0.00004	1.15	0.70105	1.61	0.04623	2.00	0.00988
Potassium uptake protein, integral membrane component, KtrB	7.74	0.00006	10.81	0.00002	50.92	0.02191	71.15	0.01638	-0.15	0.15959
Putative conjugal transfer protein MobA/MobL	183.02	0.00023	9.49	0.01454	8.09	0.08695	-2.39	0.44355	22.63	0.02615
Pyruvate-flavodoxin oxidoreductase	-0.52	0.05848	-0.92	0.52386	-0.36	0.01504	-0.65	0.09510	1.42	0.16352
RNA polymerase sigma factor for flagellar operon	-0.76	0.02253	1.93	0.00032	-0.82	0.08962	2.08	0.00013	-0.93	0.40060

[a] - All samples were run in triplicate and *gyrA* used for normalization.
