# Supplementary material for: Human Gut Symbiont Roseburia hominis Promotes and Regulates Innate Immunity
Source: Front Immunol. 2017 Sep 26;8:1166. doi: 10.3389/fimmu.2017.01166 (PMC5622956; doi:10.3389/fimmu.2017.01166)
Supplement: Supplementary file 7 [file Table_3.PDF]

Table S3. Immune system response pathway analysis: transcripts differentially expressed in the ascending colon between the *R. hominis*-treated and GF mice at day 28.

**Regulatory processes/Immune system response pathway**	***P*-value***	**Significant****	**Total*****
Immune response_IL-10 signaling pathway	0.00125	10	26
Immune response_IL-9 signaling pathway	0.00592	11	36
Immune response_HMGB1/RAGE signaling pathway	0.00832	14	53
Immune response_BCR pathway	0.00992	14	54
Development_GM-CSF signaling	0.01258	13	50
Development_PEDF signaling	0.02618	12	49
Immune response_IL-5 signalling	0.02840	11	44
Immune response_TCR and CD28 co-stimulation in activation of NF-kB	0.03611	10	40
Immune response_Regulation of T cell function by CTLA-4	0.04598	9	36
Immune response_CD40 signaling	0.04796	14	65
Signal transduction_JNK pathway	0.04921	10	42

*** -** Differentially expressed genes ($P<0.05$) were imported into the GeneGo MetaCore analytical software to determine significantly enriched canonical pathways in each group.

** - The number of genes on each map with significant differential expression between specific treatments.

*** - The total number of genes on each map.
