# Supplementary material for: Human Gut Symbiont Roseburia hominis Promotes and Regulates Innate Immunity
Source: Front Immunol. 2017 Sep 26;8:1166. doi: 10.3389/fimmu.2017.01166 (PMC5622956; doi:10.3389/fimmu.2017.01166)
Supplement: Supplementary file 8 [file Table_4.PDF]

Table S4. Immune system response pathway analysis: transcripts differentially expressed in the ascending colon between the TLR5 KO and WT mice mono-colonized with *R. hominis*.

#	Maps	*P*-value*	Significant**	Total***
1	Immune response_IL-9 signaling pathway	0.00001	7	36
2	Immune response_Histamine signaling in dendritic cells	0.00008	7	50
3	Immune response_HMGB1/RAGE signaling pathway	0.00012	7	53
4	Immune response_IL-6 signaling pathway	0.00046	5	31
5	Immune response_Histamine H1 receptor signaling in immune response	0.00052	6	48
6	Immune response_Oncostatin M signaling via MAPK in mouse cells	0.00082	5	35
7	Immune response_Oncostatin M signaling via MAPK in human cells	0.00107	5	37
8	Signal transduction_JNK pathway	0.00191	5	42
9	Immune response_IL-7 signaling in B lymphocytes	0.00213	5	43
10	Immune response_Signaling pathway mediated by IL-6 and IL-1	0.00362	4	30
11	Development_GM-CSF signaling	0.00415	5	50
12	Immune response_T cell receptor signaling pathway	0.00492	5	52
13	Chemotaxis_Leukocyte chemotaxis	0.00528	6	75
14	Immune response_CCL2 signaling	0.00579	5	54
15	Immune response_CD28 signaling	0.00579	5	54
16	Immune response_Role of DAP12 receptors in NK cells	0.00579	5	54
17	Immune response_Fc epsilon RI pathway	0.00626	5	55
18	Immune response_Role of PKR in stress-induced antiviral cell response	0.00728	5	57
19	Immune response_HMGB1 release from the cell	0.01114	4	41
20	Immune response_IL-15 signaling	0.01176	5	64
21	Immune response_HTR2A-induced activation of cPLA2	0.01313	4	43
22	Immune response_IL-4 signaling pathway	0.01421	4	44
23	Immune response_IL-5 signalling	0.01421	4	44
24	Immune response_Fc gamma R-mediated phagocytosis in macrophages	0.01653	4	46
25	Immune response_NF-AT signaling and leukocyte interactions	0.01653	4	46
26	Development_PEDF signaling	0.02043	4	49
27	Immune response_IL-2 activation and signaling pathway	0.02043	4	49
28	Immune response_NFAT in immune response	0.02332	4	51
29	Immune response_IL-3 activation and signaling pathway	0.02803	3	31
30	Immune response_BCR pathway	0.02809	4	54
31	Immune response_TLR signaling pathways	0.02809	4	54
32	Immune response_Immunological synapse formation	0.03727	4	59
33	Immune response_Th17 cell differentiation	0.03836	3	35
34	Immune response_Human NKG2D signaling	0.04722	3	38

* - Differentially expressed genes ($P<0.05$) were imported into the GeneGo MetaCore analytical software to determine significantly enriched canonical pathways in each group.

** - The number of genes on each map that are differentially expressed in the specific treatment comparison.

*** - The total number of genes on each map.
