# Supplementary material for: Human Gut Symbiont Roseburia hominis Promotes and Regulates Innate Immunity
Source: Front Immunol. 2017 Sep 26;8:1166. doi: 10.3389/fimmu.2017.01166 (PMC5622956; doi:10.3389/fimmu.2017.01166)
Supplement: Supplementary file 9 [file Table_5.PDF]

Table S5. The *p*-values for weight differences on days treated with DSS or DSS + *R. hominis*.

Day	DSS vs control	Rh/DSS vs control	Rh/DSS v DSS
10	0.96	0.90	0.68
11	0.48	0.59	0.97
12	0.37	0.18	0.88
13	0.00	0.01	0.59
14	<0.001	0.00	0.04
15	<0.001	<0.001	<0.001

Body weight at 8 days (prior to start of DSS): 20±2g. DSS: free access to 30g/l in drinking water. Mice given DSS or DSS + *R. hominis* had similar food and water intakes (p=0.81 and 0.95, respectively).
