# Supplementary material for: Human Gut Symbiont Roseburia hominis Promotes and Regulates Innate Immunity
Source: Front Immunol. 2017 Sep 26;8:1166. doi: 10.3389/fimmu.2017.01166 (PMC5622956; doi:10.3389/fimmu.2017.01166)
Supplement: Supplementary file 10 [file Data_Sheet_1.PDF]

SI Materials and Methods

Bacterial growth conditions

R. hominis A2-183^T (=DSM 16839^T=NCIMB 14029^T) was grown anaerobically using synthetic YCFA (Duncan et al., 2006) or complex M2GSC (Miyazaki et al., 1997) media. Culture was inoculated from a frozen stock into the Hungate tubes and incubated overnight at 37˚C. Bacteria were then grown on M2GSC agar plates for 48h in a MACS-MG-1000 anaerobic workstation (Don Whitley Scientific) under the atmosphere of 80% N_2, 10% CO_2, and 10% H_2 at 37˚C. The effect of mucin was investigated by adding 0.5% (w/v) mucin from porcine stomach type III (Sigma-Aldrich) to the YCFA medium.

For colonization of germfree (GF) mice, *R. hominis* was anaerobically grown in YCFA media overnight at 37˚C. The cells were harvested by centrifugation (3,500x*g*, 5 min) and the pellet was resuspended in one mL of YCFA medium, supplemented with 2% cysteine (w/v, Sigma-Aldrich) and 3% ascorbic acid (w/v, Sigma-Aldrich) for protection against oxygen.

Animal experiments

C3H/HeN and C57BL/6 mice were purchased from Harlan Laboratories. Mice were housed within HEPA-filtered flexi-film isolators (Bell Isolation Systems) at the University of Aberdeen. GF C3H/HeN were provided and maintained in the INRA gnotobiotic rodent breeding facility at Jouy-en-Josas (ANAXEM platform, Institut Micalis, INRA, Jouy-en-Josas, France). Germfree TLR5KO and wild type C57BL/6 were provided by Andrew Gewirtz (Center for Inflammation, Immunity, and Infection and Department of Biology, Georgia State University, Atlanta, GA 30303, USA) and maintained in the INRA gnotobiotic rodent breeding facility at Jouy-en-Josas. Boy/J (C57BL/6 B6 Cd45.1) mice were the background controls for conventional

TLR5K0. They carry a CD45.1 pan leukocyte marker but are otherwise equivalent to C57BL/6 wild-type mice (Flores-Langarica et al., 2012; Zhan et al., 2016). The management and experimental procedures were approved by the respective Local Ethical Review Committees.

Eighteen GF C3H/HeN male mice were allocated into control (N=8) and treatment (N=10) groups and caged individually in plastic isolators. The mice were fed *ad libitum* a sterilized commercial diet (R03-40; UAR). At day 0, 1 and 2, animals in the treatment group were given 100 µL (10^9 CFU) of *R. hominis* culture by gavage, while control animals were given 100 µL YCFA media. At days 14 and 28, four control animals and five *R. hominis*-treated animals were sacrificed. The ileum and ascending colon were divided into four equal parts and transferred to RNAlater (Ambion), neutral buffered formalin (NBF; Sigma-Aldrich) or liquid nitrogen. The whole caecum was transferred to RNAlater.

Three GF TLR5KO mice and three C57BL/6 WT mice were inoculated with *R. hominis* culture as described above to evaluate the functional importance of *R. hominis* flagellins. On day 28 the animals were sacrificed together with their GF control counterparts.

Microarray analyses

Bacterial microarray

Bacterial RNA was isolated from the mouse caecum contents using the RNeasy Mini Kit (Qiagen), and further processed with the MICROB*Enrich*™ Kit (Ambion), the MICROB*Express*™ Bacterial mRNA Enrichment Kit (Ambion), and the MessageAmp™ II-Bacteria RNA Amplification Kit (Applied Biosystems). RNA was labeled with either dCTP-Cy3 or dCTP-Cy5 during cDNA synthesis (CyScribe First Strand cDNA Labelling Kit; Amersham). Labeled products were purified using the CyScribe GFX Purification Kit (Amersham). PCR products amplified from 6000 clones in the *E. coli* plasmid RA8 library of *R. hominis* were arrayed

in duplicate on aminosilane-coated microscope slides (Corning) using a MicroGrid II TAS (BioRobotics). Amplified fragments of the housekeeping genes *rpoD* and *gyrA* were randomly distributed on the array as controls. Microarray hybridization was performed in the GeneTAC hybridization station (Genomic Solutions). Dye labeling was swapped for a second hybridization, and a separate RNA purification was also labeled and hybridized twice, to ensure reproducibility and to obtain statistically significant results. In total, four slides were hybridized for each comparison, for a total of 12 hybridizing spots per amplified clone. Fluorescence was measured in two channels using a GeneTAC LS IV (Genomic Solutions) with GeneTac Integrator version 3.0.1 software. Spot intensities were log-transformed and Loess normalization was applied to remove differences in probe labeling and hybridization efficiencies. The one-sample t-test was used on the log-ratio values to test for differential expression. Results were considered significant with more than two-fold and $p<0.05$.

Mouse microarray analysis

Ileal and ascending colonic tissues were removed from RNAlater and lyzed in Trizol (Invitrogen). RNA was isolated using the standard chloroform/isopropanol steps. Total RNA was further purified with the RNeasy Kit (Qiagen), including an RNase-free DNase I (Qiagen) digestion step. RNA integrity was determined using the Agilent 2100 Bioanalyzer (Agilent Technologies). Total RNA was processed into biotin-labeled cRNA using the One-Cycle Target Labeling Kit (Affymetrix) or biotin-labeled aRNA using the 3' IVT Express Kit (Affymetrix). Hybridization to the GeneChip NuGO Mouse Array and GeneChip Mouse Genome Array (Affymetrix) on a GeneChip Fluidics Station 450 (Affymetrix) was performed at the Institute of Medical Sciences Microarray Core Facility (University of Aberdeen, UK).

Chips were scanned with an Affymetrix GeneChip Scanner 3000 (Affymetrix). Image quality analysis was performed using the Gene Chip Operating Software (GCOS) (Affymetrix). Further data analysis was performed with the freely available software packages R (http://www.r-project.org) and Bioconductor (http://www.bioconductor.org). The moderated F-test provided by the Bioconductor package limma was used to test for differential expression. The difference was considered significant with $P<0.05$ using the Benjamini and Hochberg false discovery method. Statistical analysis was performed separately for each of the two time-points.

All differentially expressed genes ($p<0.05$) were imported into the MetaCore analytical software (GeneGo, St Joseph, MI) to generate pathway maps. Integrated pathway enrichment analysis was performed using the knowledge-based canonical pathways and endogenous metabolic pathways. Ranking of the relevant integrated pathways was based on *P*-values calculated using hypergeometric distribution. *P*-values represented the probability of a given number of genes from the input list to match a certain number of genes in the map by chance, considering the numbers of genes in the experiment versus the number of genes in the map within the full set of all genes on maps.

Gene Ontology (GO) based functional interpretation of the data was performed using DAVID (http://david.abcc.ncifcrf.gov), an expanded version of the original web-accessible program (Dennis et al., 2003). Significantly different transcripts ($P<0.05$) were allocated into the GO category ‘Biological Process’ to unearth patterns of gene expression significantly enriched for specific GO terms.

Microarray data were submitted to the National Center for Biotechnology Information (NCBI) Gene Expression Omnibus (accession number GSE25544; http://www.ncbi.nlm.nih.gov/geo).

RT-PCR analysis

PCR primers for bacteria were designed using the on-line tool Primer3Plus (Untergasser et al., 2007) and purchased from Sigma-Aldrich. The *R. hominis*-specific primers 5'-CCCACTGACAGAGTATGTAATGTAC-3' and 5'-GCACCACCTGTCACCAC-3' were used for semi-quantitative and qPCR analyses of faecal DNA samples to validate gut colonization levels. The qPCR RT-qPCR analyses were performed using a 7500 Fast Real-Time PCR System (Applied Biosystems) with the Power SYBR Green PCR Master Mix (Applied Biosystems). The cycling step was performed as follows: one cycle at 95°C for 10 min, followed by 40 cycles at 95°C for 15 sec and 60°C for 1 min, ending with a dissociation step. All samples were run in triplicate. Expression of the *gyrA* gene was used as a housekeeping gene/transcript for normalization (Tables S1 and S2).

For host gene expression, 2 µg of total eukaryotic RNA, isolated from the ileum and ascending colon, was reverse-transcribed into cDNA using the High Capacity cDNA Reverse Transcription Kit (Applied Biosystems) with random primers. This was followed by qPCR using a 7500 Fast Real-Time PCR System (Applied Biosystems) with the QuantiFast SYBR Green PCR Kit (Qiagen) and QuantiTect Primer Assays (Qiagen). PCR cycling conditions were as follows: one cycle at 95 °C for 5 min, followed by 40 cycles at 95 °C for 10 s and at 60 °C for 30 s, ending with a dissociation step. All samples were run in triplicate. *Hprt* was used for the normalization purposes.

All RT-qPCR data were analyzed on a logarithmic scale with base 2 by one-way ANOVA with a significance cut-off of $P<0.05$. Differences were back-transformed to calculate the fold changes.

Western blot

Immuno-purified rabbit polyclonal antibodies against *R. hominis* FlaA1 and FlaA2 were produced as described by Duck et al. (2007). In brief, New Zealand white female rabbits were immunized with the synthetic peptides designed based on the corresponding sequences in the genome of *R. hominis* (Travis et al., 2015). Two peptides for each flagellin in complete Freund's adjuvant were used for immunization and for the subsequent boosts three times. For *R. hominis* FlaA1, peptide NH2-CRSQVRGLNKASDNA-CONH2 and peptide NH2-IDGNFTSKKLQVGSLC-COOH were used, while for *R. hominis* FlaA2, peptide C-AQYNDDAKSVLEILK-COOH and peptide C-GLNKASRNSQDGIS-CONH2 were used. Following immunization, the antibodies were purified on an immunoaffinity column prepared by coupling the peptides to 1 mL of activated sepharose beads.

For Western blot, *R. hominis* was grown in the presence of varying amounts (0.01 g to 1.0 g diet/10 mL of culture) of UV irradiated standard mouse chow for 3h, filtered to remove dietary components and diluted in Laemmli buffer containing 8M urea. 30 µL of each sample was loaded into the wells of a NuPAGE® Novex® 4-12% Bis-Tris gel (Invitrogen) and electrophoresed, followed by further processing using the WesternBreeze Chromogenic Immunodetection System (Invitrogen). FlaA1 and FlaA2 antibodies were diluted 1:1000 and the loading control anti-DNA gyrase A (Abcam) was diluted 1:300 in the antibody diluent. Following the incubation with antibodies at 4 °C overnight, the membrane was incubated for 1h at room temperature with alkaline phosphatase conjugated with goat anti-rabbit antibodies. The presence of antigens was detected by substrate colour development relatively to loading control colour development.

FISH analyses

Tissues fixed in neutral buffered formalin were embedded in Technovit 8100 (Heraeus Kulzer). Two-micron sections were cut using a rotary microtome (Leica/Reichert Autocut). Three

sections were taken per slide at 100 μm, 200 μm and 300 μm into the tissue, resulting in nine sections per animal.

Slides were dehydrated by consecutive incubations in 50% (v/v), 80% and 96% ethanol and dried at room temperature (RT). The 16S rRNA FISH probes used were a general bacterial probe Eub338 (GCTGCCTCCCGTAGGAGT; Cy3-labeled) and a newly designed *R. hominis* A2-183-specific probe (GTACATTACATACTCTGTCAGTG; FITC-labeled). The latter probe was extensively tested for specificity against a panel of intestinal bacterial isolates including the closest relatives, *Roseburia inulinivorans* and *Roseburia intestinalis*. Ten microliters of a probe (30 ng/μL) in 100 μL of hybridization buffer was applied to the dehydrated sample and incubated at probe-specific temperature. The slides were washed in washing buffer at 50˚C for 30 min, dipped in ice-cold water to remove residual washing buffer and dried under compressed air flow. Counterstaining was performed with 4',6-diamidino-2-phenylindole (DAPI; Vector Laboratories Inc) and slides were mounted with Vectashield Mounting Medium for fluorescence (Vector Laboratories Inc) to prevent fading. Bacteria were visualized using a Leica DM RBE fluorescence microscope (Leitz GMBH) and photographed with a Penguin 600CL camera (Pixera) and Viewfinder 3.0 software (Studio Lite). High-magnification images (x630) were retrieved using the Apochromatics system (Leica).

Immunocytochemistry

Immuno-localization of *R. hominis* flagellins was examined in the colon content of mice colonized with *R. hominis* using specific antisera raised against defined peptide sequences from both FlaA1 and FlaA2 flagellins. Gut contents were diluted in PBS, smeared on glass slides and air dried. Smears were fixed in pre-cooled methanol for 5 min at -20 °C, incubated with anti-FlaA1

or anti-FlaA2 rabbit antisera (1:125, CovaLabs) overnight at 4 °C and visualized using Alexa donkey anti rabbit 488 (1:1000, Molecular Probes).

Sections were fixed in pre-cooled methanol for 30 min at -20 ºC. Immuno-localization of T cell markers was examined on sequential cryosections (8µm). Sections were fixed either in pre-cooled methanol for 30 min at -20 ºC (Ly6G FITC, CD3 FITC, at 1:50 (BD Biosciences)), or, for the double-labeled FoxP3 Alexa Fluor 594 (1:500, Abcam) with CD3 FITC (1:100, BD Biosciences), fixed in 1% paraformaldehyde (PFA) for 2 min at RT followed by 3 min in 0.01% Triton X in PBS. All sections were blocked with 10% BSA (Sigma) containing 10% relevant pre-immune sera in PBS (pH 7.4). Methanol-fixed tissues were incubated with primary antibodies for 1h at RT. PFA-fixed sections were incubated with antibodies overnight at 4 °C. FoxP3 was visualized using Alexa goat anti rabbit 594 (1:1000, Molecular Probes). Sections were counter labeled with DAPI and mounted with Vectashield (Vector Laboratories). For quantification of positive cells, a minimum of five fields of view from each mouse section was examined, using imaging software and microscope settings described above.

Histology

Ascending colon tissue samples were fixed for three hours in neutral buffered formalin (Sigma) at RT with constant agitation. The samples were rinsed with PBS and then transferred to 70% ethanol and stored at RT until orientated for transverse sectioning and embedded in cold-curing resin using Technovit 8100 (Heraeus Kulzer) according to the manufacturer's instructions. The embedded tissue was mounted onto Histoblocs using Technovit 3040 (Heraeus Kulzer). Four micron sections were cut using a rotary microtome (Leica Autocut) fitted with a glass knife (TAAB Laboratories Equipment Ltd.). Tissue sections were stained using the standard haemotoxylin/eosin methods. A complete transverse cross sectional area of the ascending colon from each animal was

imaged at x200 magnification on a Zeiss Axioskop microscope using a QImaging camera controlled by Image Pro Plus software. Each field of view was then scored from 0 to 4 according to the method based on Berg et al. (1996). Histopathology scores were: 0 = Shallow crypts, no or few infiltrating inflammatory cells, intact epithelium, goblet cells appear full of mucin (no pathology); 1 = Crypts may exhibit slight epithelial cell hyperplasia, some diffuse infiltrating inflammatory cells may be seen between crypts, luminal epithelium appears intact, goblet cells may appear slightly depleted of mucin; 2 = Crypts appear deeper with distinct evidence of epithelial hyperplasia, depletion of mucin from goblet cells, infiltrating inflammatory cells evident and may be multifocal in nature, although infiltrates are not seen in the submucosa; 3 = Lesions involve a larger area of the mucosa and /or are more frequent than seen in grade 2. Lesions do not involve the submucosa. Luminal epithelial cells exhibit small erosions. The lesions are not transmural; 4 = Crypt epithelium appears eroded. Abscesses may be present. Luminal epithelial cells appear irregular, sometimes with complete loss. Transmural infiltrate is observed - often associated with complete loss of epithelial cells into the lumen. The mean percentage of fields of view at a given grade was calculated and treatment groups were compared using Student's t-test.

Cloning, purification and examination of recombinant flagellins

The full open reading frames (ORFs) of flagellin genes from *R. hominis*, *S.* Enteritidis and *E. coli* K12 were amplified using PCR primers targeting the corresponding genome regions. The gel-purified amplicons from *R. hominis* were inserted into the expression vector pT7-MAT-Tag-FLAG2 (Sigma) and the amplicons from *S.* Enteritidis and *E. coli* K12 were cloned into the expression vector pGEX-6P-1 (GE Healthcare). Recombinant flagellins were expressed by transformation of recombinant plasmids into *E. coli* BL21 Rosetta and *E. coli* BL21 (DE3) cells, respectively, and by induction with 1mM IPTG (isopropyl b-D-galactosidase). Recombinant

flagellins of *R. hominis* were recovered from the cell lysates of *E. coli* with TALON Metal Affinity Resin (Clonetech, Takara) and Anti-FLAG M2 Magnetic beads (Sigma) according to the manufacturer's instructions. Glutathione Agarose (Sigma) and Ni-NTA (nickel-nitriloacetic) beads (Clonetech, Takara) were used for purification of recombinant *S*. Enteritidis and *E. coli* K12 flagellins, according to the manufacturer's instructions. The purity of proteins was assessed by SDS-PAGE stained with Coomassie Blue.

Pro-inflammatory activities of flagellins were determined by the Luciferase Assay (Promega) according to the manufacturer's instructions using the NF-κB transformed Caco-2 cell line. Caco-2 cells were incubated with recombinant flagellins at a final concentration of 100 ng/μL for 2h at 37°C in a 75% humidified atmosphere of 5% CO2. After the treatment, the cells were washed with PBS solution twice and harvested for total RNA isolation.

Intestinal Epithelial Cell experiments

All cell culture reagents, unless specified otherwise, were supplied by Sigma-Aldrich. For cell culture experiments with recombinant flagellins, $5x10^4$ Caco-2 cells were seeded in 24-well plates in DMEM (high glucose, HEPES) medium supplemented with heat-inactivated fetal bovine serum (Gibco), penicillin, streptomycin, amphotericin B and L-glutamine at 37°C in a 75% humidified atmosphere of 5% CO2. The cells reached confluence on day 5-6 and were used 3 days post-confluency. Prior to any treatment, cells were washed twice with Hanks' Balanced Salt Solution and kept in DMEM supplemented with L-Glutamine, selenium and transferrin for 24 hours.

Isolation of intestinal and MLN cells

Cells from the small intestine and mesenteric lymph nodes (MLN) were isolated as previously described, with minor modifications (Monteleone et al., 2008). Briefly, cellular suspensions were incubated with 100U/ml collagenase VIII (Sigma-Aldrich) in RPMI supplemented with 20% FBS at 37°C for 20min (mesenteric lymph nodes) or 1 hour (small intestine tissue). The separated cell suspensions were then analyzed by flow cytometry.

Generation of bone marrow-derived dendritic cells and cultures

Bone marrow cells were obtained from the femur and tibia of C3H/HeN and C57Bl/6 mice as previously described (Inaba et al., 1992; Brasel et al., 2000; Weigel et al., 2002; Xu et al., 2007). For GMCSF-derived dendritic cells, bone marrow cells were resuspended at 1x10^{6}/mL in RPMI supplemented with 10% FCS and 20ng/mL rmGM-CSF and seeded at 10mL/plate in 100mm^{2} tissue culture plates. After three days of culturing, the loosely adherent cells were collected and replated with GM-CSF supplemented media at 1x10^{6}/mL in 12 well tissue culture plates. At day 5, cells were stimulated with 100ng/mL flagellins before being harvested on day 6. For Flt3L-derived dendritic cells, bone marrow cells were resuspended at 2x10^{6}/mL in RPMI supplemented with 10% FCS and 200ng/mL rmFlt3 and seeded at 2mL/well in 12-well tissue culture plates. Cells were cultured for 10 days with an additional 2mL of Flt3 media added to each well on day 4. At day 9, cells were stimulated with 100ng/mL flagellins before being harvested on day 10. Cells were harvested from plates by gentle pipetting and analyzed by flow cytometry.

Purification of OTII TCR transgenic CD4 T cells and DC antigen-presentation cultures

Male OTII transgenic mice were killed, and their peripheral lymph nodes and spleens were harvested to prepare cell suspensions. CD4+ T cells were purified using MACS Mitenyi CD4 T cell isolation kits that purify CD4 T cells using a negative-selection approach. This purification

protocol routinely yielded highly pure (>95% pure) CD4+T cell populations as judged by FACS analysis (data not shown). For mixing studies, Flt-3 derived BMDC (5×10^4 cells) were cultured and stimulated with 100ng/ml flagellins for 24 hrs, washed to remove flagellins and reseeded purified CD4+ T cells (2×10^5 cells) and OVA323–339 peptide (1 μg/ml) in wells of a 96-well, round-bottom plate. After 48 h incubation at 37°C in 5% CO_2, cells were isolated and co-culture T cell population characterised by flow cytometry

Flow Cytometry

Single-cell suspensions of lamina propria, mesenteric lymph node and dendritic cells were incubated in blocking buffer (containing serum and CD16/CD32 antibody) at 4°C for 15 min prior to staining with specific fluorochrome-conjugated antibodies. Lamina propria cells were labeled with antibodies to murine CD4-FITC and CD25-APC (eBioscience), CD8-APC-Cy7, CD3-PerCP Cy5.5 and B220-BV570 (Biolegend). Intracellular FoxP3-PE (eBioscience) labelling was performed after extracellular staining and cell fixation/permeabilization according to the manufacturer's instructions (eBioscience). GM-CSF-derived dendritic cells were labeled with antibodies CD11b-PerCP Cy5.5 (BD Biosciences), CD11c-PE-Cy7, I-A/I-E-APC-Cy7, CD80-PE, CD86-APC, CD8-FITC, B220-BV570 (Biolegend). Flt3L-derived dendritic cells were labelled with CD11c-PE-Cy7, CD11b- or Siglec-H-PerCP Cy5.5 (Biolegend), I-A/I-E-APC-Cy7, CD317-PE, CD40-Alexa Fluor 647, CD103-FITC and B220-BV570. Co-culture T cells were labelled with CD4-PerCP Cy5.5 (BD Biosciences), CD127-PE-Cy7, CD73-APC, CD195 (CCR5)-FITC, CD62L-BV570 and CD25-APC-Cy7 (Biolegend). Cells were analyzed using a FACSAriaII (BD Biosciences) and FlowJo software version 7.2.5.

Cytometric Bead Array (CBA)

Bone marrow cells were isolated from the femur and tibia of C3H/HeN and C57Bl/6 mice and Flt3L-expanded in RPMI media as described previously (Inaba et al., 1992; Brasel et al., 2000; Weigel et al., 2002; Xu et al., 2007). Cells were stimulated with 100 ng/mL RH1 after 9 days of culture, and the supernatant was collected on day 10. The experiment was performed on three separate occasions to generate N=3.

CBA analysis was performed on cell supernatants using the Cytometric Bead Array Mouse Enhanced Sensitivity Master Buffer Kit (BD Biosciences) according to the manufacturer's instructions. Standards and samples were loaded onto a 96-well plate for measurement in a FACSArray (BD Biosciences). Results were analyzed using BD FCAP software (BD Biosciences).

Dry body weight and lipid carcass analysis

Eviscerated mouse carcass was weighed, lyophilized to constant weight and then milled for analysis. Lipid content was determined by extraction (1:100 w/v) with chloroform/methanol (2:1 v/v) as described previously (Olivera et al., 2003).

References

1. Berg DJ, Davidson N, Kuhn R, Muller W, Menon S, Holland G, Thompson-Snipes L, Leach MW, Rennick D. Enterocolitis and colon cancer in interleukin-10-deficient mice are associated with aberrant cytokine production and CD4(+) TH1-like responses. J. Clin. Invest. 1996, 98, 4, 1010-1020.
2. Brasel K, De Smedt T, Smith JL, Maliszewski CR. Generation of murine dendritic cells from flt3-ligand-supplemented bone marrow cultures. Blood 2000 Nov 1;96(9):3029-3039.

3. Dennis G, Jr, Sherman BT, Hosack DA, Yang J, Gao W, Lane HC, et al. DAVID: Database for Annotation, Visualization, and Integrated Discovery. Genome Biol 2003;4(5):P3.
4. Duck LW, Walter MR, Novak J, Kelly D, Tomasi M, Cong Y, et al. Isolation of flagellated bacteria implicated in Crohn's disease. Inflamm Bowel Dis 2007 Oct;13(10):1191-1201.
5. Duncan SH, Aminov RI, Scott KP, Louis P, Stanton TB, Flint HJ. Proposal of *Roseburia faecis* sp. nov., *Roseburia hominis* sp. nov. and *Roseburia inulinivorans* sp. nov., based on isolates from human faeces. Int J Syst Evol Microbiol 2006; 56(Pt 10):2437-2441.
6. Flores-Langarica A, Marshall JL, Hitchcock J, Cook C, Jobanputra J, Bobat S, et al. Systemic flagellin immunization stimulates mucosal CD103+ dendritic cells and drives Foxp3+ regulatory T cell and IgA responses in the mesenteric lymph node. J Immunol 2012; 189(12):5745-5754.
7. Inaba K, Inaba M, Romani N, Aya H, Deguchi M, Ikehara S, et al. Generation of large numbers of dendritic cells from mouse bone marrow cultures supplemented with granulocyte/macrophage colony-stimulating factor. J Exp Med 1992; 176(6):1693-1702.
8. Miyazaki K, Martin JC, Marinsek-Logar R, Flint HJ. Degradation and utilization of xylans by the rumen anaerobe *Prevotella bryantii* (formerly *P. ruminicola* subsp. *brevis*) B14. Anaerobe 1997; 3: 373–381.
9. Monteleone I, Platt AM, Jaensson E, Agace WW, Mowat AM. IL-10-dependent partial refractoriness to Toll-like receptor stimulation modulates gut mucosal dendritic cell function. Eur J Immunol. 2008 38(6):1533-47.
10. Olivera L, Canul RR, Pereira-Pacheco F, Cockburn J, Soldani F, McKenzie NH, et al. Nutritional and physiological responses of young growing rats to diets containing raw cowpea seed meal, protein isolate (globulins), or starch. J Agric Food Chem 2003 Jan 1;51(1):319-325.

11. Travis AJ, Kelly D, Flint HJ, Aminov RI. Complete genome sequence of the human gut symbiont *Roseburia hominis*. Genome Announc. 2015; 3(6). pii: e01286-15.

12. Untergasser A, Nijveen H, Rao X, Bisseling T, Geurts R, Leunissen JA. Primer3Plus, an enhanced web interface to Primer3. Nucleic Acids Res 2007 Jul;35(Web Server issue):W71-4.

13. Weigel BJ, Nath N, Taylor PA, Panoskaltsis-Mortari A, Chen W, Krieg AM, et al. Comparative analysis of murine marrow-derived dendritic cells generated by Flt3L or GM-CSF/IL-4 and matured with immune stimulatory agents on the *in vivo* induction of antileukemia responses. Blood 2002 Dec 1;100(12):4169-4176.

14. Xu Y, Zhan Y, Lew AM, Naik SH, Kershaw MH. Differential development of murine dendritic cells by GM-CSF versus Flt3 ligand has implications for inflammation and trafficking. J Immunol 2007 Dec 1 ;179(11):7577-7584.

15. Zhan T, Cao C, Li L, Gu N, Civin CI, Zhan X. MIM regulates the trafficking of bone marrow cells via modulating surface expression of CXCR4. Leukemia 2016;30 (6): 1327-34.
